# Homeostasis in the soybean miRNA396–*GRF* network is essential for productive soybean cyst nematode infections

**DOI:** 10.1093/jxb/erz022

**Published:** 2019-01-30

**Authors:** Jason B Noon, Tarek Hewezi, Thomas J Baum

**Affiliations:** 1 Department of Plant Pathology and Microbiology, Iowa State University, Ames, IA, USA; 2 Department of Plant Sciences, University of Tennessee, Knoxville, TN, USA

**Keywords:** Growth-regulating factors, *Heterodera glycines*, miRNAs, miRNA396, plant-parasitic nematodes, post-transcriptional regulation, soybean, soybean cyst nematode, syncytium

## Abstract

*Heterodera glycines*, the soybean cyst nematode, penetrates soybean roots and migrates to the vascular cylinder where it forms a feeding site called the syncytium. MiRNA396 (miR396) targets *growth-regulating factor* (*GRF*) genes, and the miR396–*GRF1/3* module is a master regulator of syncytium development in model cyst nematode *H. schachtii* infection of Arabidopsis. Here, we investigated whether this regulatory system operates similarly in soybean roots and is likewise important for *H. glycines* infection. We found that a network involving nine *MIR396* and 23 *GRF* genes is important for normal development of soybean roots and that *GRF* function is specified in the root apical meristem by miR396. All *MIR396* genes are down-regulated in the syncytium during its formation phase while, specifically, 11 different *GRF* genes are up-regulated. The switch to the syncytium maintenance phase coincides with up-regulation of *MIR396* and down-regulation of the 11 *GRF* genes specifically via post-transcriptional regulation by miR396. Furthermore, interference with the miR396–*GRF6/8–13/15–17/19* regulatory network, through either overexpression or knockdown experiments, does not affect the number of *H. glycines* juveniles that enter the vascular cylinder to initiate syncytia, but specifically inhibits efficient *H. glycines* development to adult females. Therefore, homeostasis in the miR396–*GRF6/8–13/15–17/19* regulatory network is essential for productive *H. glycines* infections.

## Teaser

A network of nine *MIR396* and 11 *GRF* genes delineates development of the crucial soybean cyst nematode feeding site, and homeostasis is necessary for successful infections.

## Introduction

Cyst nematodes (*Heterodera* and *Globodera* spp.) are economically important, root-infecting, obligate biotrophs that form an elaborate feeding site within the vascular cylinder called the syncytium (Hussey and Grundler, 1998; Jones *et al.*, 2013). The syncytium serves as the single source of nourishment throughout the life of the cyst nematode. The development of the feeding organ is initiated by migratory pre-parasitic second-stage juveniles (pre-J2) that enter the vascular cylinder and select a single cell that becomes enlarged and multinucleated [during parasitic (par)-J2 and early J3 stages] through cytoplasmic fusion of numerous nearby cortical or vascular parenchyma cells via cell wall dissolution. Syncytia are characterized by dense cytoplasm, reduced vacuoles, hypertrophied nuclei and nucleoli, and abundant endoplasmic reticulum, ribosomes, plastids, and mitochondria (Sobezak and Golinowski, 2009). This process of redirecting differentiated root cells into a novel developmental program ensues during a syncytium formation phase that involves immense transcriptional and post-transcriptional regulation of gene expression (Alkharouf *et al.*, 2006; Ithal *et al.*, 2007*b*; Klink *et al.*, 2009; Szakasits *et al.*, 2009; Hewezi and Baum, 2012; Hewezi *et al.*, 2012). The fully formed syncytium then enters a maintenance phase (late in the J3 stage) where no additional cells are incorporated and, thus, has completed all major developmental changes for maintaining the function of feeding the developing nematode. Interestingly, much of this reprogramming of differentiated root cells involves the concerted action of small RNAs, in particular miRNAs and their target genes (Hewezi *et al.*, 2008, 2012; Hewezi and Baum, 2012, 2015).

In plants, miRNAs are 20–24 nt endogenous molecules that are produced from their own *MIRNA* genes and function to suppress gene expression (Rogers and Chen, 2013). *MIRNA* genes are transcribed and produce a primary miRNA transcript that is first processed by DICER-LIKE 1 (DCL1) into a precursor (pre)-miRNA stem–loop structure (Bologna and Voinnet, 2014). The pre-miRNA is subsequently processed by DCL1 (if a 21 nt miRNA) into short dsRNAs consisting of miRNA guide and passenger (miRNA*) strands (Bologna and Voinnet, 2014). The miRNA/miRNA* duplex is 2'-*O*-methylated at the 3' ends for stabilization (Yu *et al.*, 2005). Then, most commonly, the miRNA guide strand is loaded into the ARGONAUTE (AGO) component of the RNA-induced silencing complex (RISC) (Eamens *et al.*, 2009). miRNA-loaded RISCs are then directed to target transcripts through miRNA/target complementarity and repress target transcripts most often through slicing or cleavage via AGO endonuclease activity (Mallory *et al.*, 2008). miRNAs regulate the expression of transcription factors, proteins that mediate stress responses, and many other proteins that impact the development and physiology of plants (Rogers and Chen, 2013).

Various miRNAs change in expression in response to cyst and root-knot nematode infection (Hewezi *et al.*, 2008; Li *et al.*, 2012; Xu *et al.*, 2014; Hewezi and Baum, 2015; Zhao *et al.*, 2015; Cabrera *et al.*, 2016). During infection of Arabidopsis by the beet cyst nematode *Heterodera schachtii*, many miRNAs are differentially expressed and are negatively correlated with target gene abundance (Hewezi *et al.*, 2008). Of these differentially expressed miRNAs, miR396 was shown to be a master regulator of syncytium development (Hewezi *et al.*, 2012). Also, an important role for miR390, TAS3 *trans*-acting short-interfering (tasi) RNAs, and their auxin response factor targets was demonstrated for root-knot nematode *Meloidogyne javanica* infection of Arabidopsis (Cabrera *et al.*, 2016). Furthermore, the miR319–TCP4 module was shown to act as a responder and regulator of systemic defense signals, mediated by jasmonic acid, for resistance to the root-knot nematode *Meloidogyne incognita* in tomato (*Solanum lycopersicum*) (Zhao *et al.*, 2015). These previous findings demonstrate that miRNAs are important regulatory factors during infection by cyst and root-knot nematodes (Hewezi and Baum, 2015).

Deep sequencing efforts have revealed that miRNAs in soybean (*Glycine max*) are differentially expressed during seed development, flowering time, and in the shoot apical meristem (Wong *et al.*, 2011; Shamimuzzaman and Vodkin, 2012; Li *et al.*, 2015). Soybean miRNAs are also differentially expressed during various abiotic (Kulcheski *et al.*, 2011; Fang *et al.*, 2013; Wang *et al.*, 2013; Xu *et al.*, 2013; Liu *et al.*, 2017) and biotic stress conditions. These biotic stress conditions include rot and rust diseases (Guo *et al.*, 2011; Kulcheski *et al.*, 2011) and, interestingly, *H. glycines* infection (Li *et al.*, 2012; Xu *et al.*, 2014; Tian *et al.*, 2017). However, the latter studies revealed only limited miRNA profiles. Collectively though, these deep sequencing efforts suggest that miRNAs are involved in a wide range of important processes in soybean, including *H. glycines* infection. Soybean miRNAs have been experimentally confirmed to be important for nodulation (H. Li *et al.*, 2010; Turner *et al.*, 2013; Yan *et al.*, 2013, 2015, 2016; Wang *et al.*, 2014, 2015) and low water availability (Liu *et al.*, 2017).

miR396 targets the plant-specific growth-regulating factor (GRF) transcription factors characterized by the glutamine, leucine, glutamine (QLQ) protein interaction and tryptophan, arginine, cysteine (WRC) DNA-binding domains (Kim *et al.*, 2003). The Arabidopsis miR396–*GRF* regulatory module is important for many developmental and stress-related processes (van der Knaap *et al.*, 2000; Kim *et al.*, 2003; Kim and Lee, 2006; Horiguchi *et al.*, 2011; Hewezi and Baum, 2012; Hewezi *et al.*, 2012; Kim *et al.*, 2012; J. Liu *et al.*, 2012; Bao *et al.*, 2014; Debernardi *et al.*, 2014; Liang *et al.*, 2014; Liu *et al.*, 2014; Pajoro *et al.*, 2014; Rodriguez *et al.*, 2015), but the most generalized function is the regulation of cell proliferation and expansion (Kim *et al.*, 2003; Rodriguez *et al.*, 2010; Horiguchi *et al.*, 2011; Kim *et al.*, 2012; Bao *et al.*, 2014; Liang *et al.*, 2014; Pajoro *et al.*, 2014; Omidbakhshfard *et al.*, 2015; Rodriguez *et al.*, 2015). Homeostasis of the miR396–*GRF* regulatory module in both Arabidopsis and *Medicago truncatula* is important for normal root development (Hewezi *et al.*, 2012; Bazin *et al.*, 2013; Rodriguez *et al.*, 2015). Recently, overexpression of soybean miR396 precursors in Arabidopsis gave an altered root phenotype (Liu *et al.*, 2017). Interestingly, Arabidopsis *GRF1*- and *GRF-3*-mediated gene expression regulation probably accounts for almost 50% of the genes that are differentially expressed in the *H. schachtii* syncytium (Szakasits *et al.*, 2009; Hewezi *et al.*, 2012), and interfering with the miR396–*GRF1/3* regulatory module resulted in decreased syncytium size and arrested nematode development (Hewezi *et al.*, 2012). Thus, the miR396–*GRF* regulatory module may serve as a possible target for developing novel control measures against cyst nematodes.

Here we investigate whether a soybean miR396–*GRF* regulatory system is involved in, and necessary for, productive *H. glycines* infections. To the best of our knowledge, this is the first study that has attempted to extend findings made in the Arabidopsis–*H. schachtii* model system to the agronomically important interaction between soybean and *H. glycines*. Using a combination of molecular and genetic analyses, we first determine that a complex network involving nine *MIR396* genes and 23 *GRF* genes operates in soybean roots. Interference with this regulatory network modifies the lateral root system, but the amount of root tissue available for *H. glycines* infections, and the number of J2 inside the vascular cylinder early on in infection are unaffected. Later on during *H. glycines* infection, we determine that a network involving all nine *MIR396* and 11 different *GRF* genes delineates the syncytium formation phase, which begins with a miR396 down-regulation and a resulting *GRF* up-regulation. During the switch to the syncytium maintenance phase, a miR396 expression spike in the syncytium post-transcriptionally silences *GRF* genes. Furthermore, we indicate that interference with the homeostasis of this network prevents efficient *H. glycines* development to the adult female stage, showing an essential role for this regulatory network in productive *H. glycines* infections.

## Materials and methods

### Inoculation of whole plants

Soybean cultivar (cv.) Williams 82 seeds were surface sterilized with 10% sodium hypochlorite for 10 min and planted on seed germination paper (Anchor Paper). Ragdolls were incubated at 26 °C in the dark for 3 d. Seedlings were placed on circular steel blue seed germination blotter paper (Anchor paper) dampened with MES-buffered ddH_2_O, pH 6.5 in a circle with the radical tips facing towards the center (10 seedlings per plate). Each radical was inoculated with 500 surface-sterilized *H. glycines* line OP50 pre-J2s (Baum *et al.*, 2000). Inoculated radicals were covered with dampened blotter paper, and infection chambers were incubated at 26 °C in the dark for 24 h. Four inoculated seedlings were acid fuchsin stained for *H. glycines* (Hussey, 1985) to ensure adequate infections. Infected seedlings were rinsed and placed back into ragdolls and incubated in a Percival growth chamber at 26 °C with a 14:10 h light dark cycle.

### *In silico* analyses

Soybean pre-miR396 sequences from miRBase (Kozomara and Griffiths-Jones, 2014) were blastn-searched against the soybean genome at SoyBase using default parameters. Pre-miR396 stem–loops were modeled *in silico* using the Mfold Web Server (Zuker, 2003) with default settings. All GRF coding sequences (CDS) were submitted to the psRNATarget server (Dai and Zhao, 2011) with default parameters along with all miR396 molecules to evaluate for putative miR396 target sites.

### Phylogenetic analysis

Multiple sequence alignments (MSAs) were generated with Clustal using default parameters in MEGA6 (Tamura *et al.*, 2011). Poorly aligned regions were removed. Model selection analysis was performed in MEGA6 using default parameters to obtain the best-scoring model of nucleotide substitution. Phylogenetic analysis was performed in MEGA6 using bootstrapped Maximum Likelihood (ML) estimation with 100 bootstrap replications. Reported is the best scoring ML phylogenetic tree with bootstrap values indicated on the corresponding nodes.

### Assessment of syncytial phases

Roots from five plants per time point were acid fuchsin stained for *H. glycines* (Hussey, 1985). For each plant, 100 *H. glycines* were observed with a stereo microscope (Zeiss) and each life stage was recorded. Pre-J2 have a slender body and tapered tail, and are most often found migrating through the cortex, while par-J2 have a swollen body and rounded tail, and are anteriorly attached in the vascular cylinder. J3 are much more swollen than even par-J2, and the rounded tail is more pronounced. J4/adult females are much larger than even J3, and have a near lemon shape. The acid fuchsin staining with thorough clearing in acidified glycerol allowed the stages to be easily distinguished with a stereo microscope. The average percentage of each life stage was calculated for each time point. This method was also used to compare the number of *H. glycines* penetrating J2s that infected the roots (i.e. reached the vascular cylinder) of the various mutants and empty vector (EV) controls.

### RNA isolation and cDNA synthesis

Total RNA was isolated from 50 mg of ground root tissue using the NucleoSpin Kit (Clontech). Yields and purity were assessed with a NanoDrop, and integrity with agarose gel electrophoresis. Total RNA was polyadenylated and reverse transcribed using the Mir-X miRNA Kit (Clontech), which generates cDNA for both mature miRNAs and naturally polyadenylated transcripts, allowing quantitative real-time reverse transcription–PCR (qRT–PCR) analysis of pre-miR396, mature miR396, and *GRF* genes all from the same samples. cDNA was prepared from transgenic hairy root total RNA samples with qScript cDNA SuperMix (Quanta). In all cases, 1 μg of total RNA was used to prepare cDNA.

### qRT–PCR

qRT–PCR was performed with iQ SYBR Green (Bio-Rad) on an iCycler iQ Real-Time PCR Detection System (Bio-Rad). For all reactions, cDNA consisted of 1/15th of the total reaction volume. Protocol: 95 °C for 3 min, 40 cycles of 95 °C for 15 s, and 60 °C for 30 s. Universal mRQ reverse primer (Mir-X miRNA Kit) was used along with miR396-specific oligonucleotides as forward primers. Forward primers specific to each pre-miR396 subfamily were used with mRQ reverse. Pre-miR396 had to be quantified as subfamilies because primers that attempted to quantify each subfamily member individually resulted in much lower primer efficiencies and poor melting curves. miR396-specific forward primers included two adenine nucleotides on the 3' ends to ensure binding to the poly(T) region of miR396 cDNAs and not to pre-miR396 (Gutierrez *et al.*, 2009). U6 (Mir-X miRNA Kit) was used as a calibrator for normalization. For *GRF* genes, RNA levels were normalized to *GmUBQ3* (GenBank: D28123.1). All primer sets were pre-validated on serially diluted soybean root cDNA with amplification efficiencies >90%. Amplification specificities were confirmed for all by melting curve analysis and agarose gel electrophoresis. Melting curve analysis protocol: 95 °C for 1 min, 55 °C for 10 s, and a slow temperature ramp from 55 °C to 95 °C . ddH_2_O and total RNA samples were included as negative controls with no amplification. Three biological replicates and four technical replicates were always used. Relative changes in gene expression levels were quantified using the 2^−∆∆CT^ method (Livak and Schmittgen, 2001). Single statistical comparisons were made using the *t*-test, and multiple comparisons by ANOVA and Tukey–Kramer HSD post-hoc test in JMP Pro 11.

### miRNA cleavage assays

miR396 cleavage sites were mapped with the FirstChoice RNA ligase-mediated (RLM)- RACE Kit (Ambion). Total RNA at 14 days post-inoculation (dpi) was poly(A)-selected with Dynabeads (Thermo) and ligated to the 5'-RACE RNA adaptor without calf intestine alkaline phosphatase treatment. cDNA synthesis was performed using *GRF*-specific outer primers. Subsequent steps followed the manufacturer’s instructions. RLM-RACE products were cloned into pGEM-T Easy (Promega) and sequenced at Iowa State University.

### Vector construction

For promoter constructs, soybean Williams 82 genomic (g)DNA was isolated from a leaf of a 3-week-old plant according to Blin and Stafford (1976). From 1.4 kbp to 2.3 kbp of upstream regulatory DNA sequence in SoyBase was cloned for each promoter construct. PCR amplification was performed with Platinum *Taq* (Invitrogen). PCR products were cloned into pGEM-T Easy and restriction digest cloned into p4305.1 (GenBank: KT954098) (restriction enzyme sites are included on the primer sequences in Supplementary Table S1 at *JXB* online). For pre-miR396 overexpression constructs, pre-miR396 were PCR-amplified from soybean gDNA using Platinum *Taq* with primers exactly 20 nt 5' and 3' to the pre-miR396 sequences in SoyBase. PCR products were cloned into pGEM-T Easy and restriction digest cloned into the pG2XPRESS derivative of pG2RNAi2 (GenBank: KT954097); the *GUS* (β-glucuronidase) linker was restriction digested out (Noon *et al.*, 2016). For RNAi, we PCR-amplified nucleotides 1–333 of the GRF9 CDS (*GRF9i*_*1–333*_) from soybean cDNA with Platinum *Taq*. PCR products were cloned into pGEM-T Easy and restriction digest cloned into pG2RNAi2 sense and antisense sites. For *rGRF9* overexpression, we PCR-amplified the *GRF9* CDS from cDNA, and synonymous mutations were introduced in the miR396 target site by overlap extension PCR (Ho *et al.*, 1989). The *rGRF9* PCR product was cloned into pGEM-T Easy and restriction digest cloned into pG2XPRESS. All empty vectors were sequenced, and then transformed into *Agrobacterium rhizogenes* strain K599.

### Hairy root nematode infection assays

Transgenic hairy roots were generated and inoculated with surface-sterilized *H. glycines* (250 pre-J2s per root tip) similar to as described previously (Baum *et al.*, 2000; S. Liu *et al.*, 2012; Noon *et al.*, 2016), but with some modifications. We used an inoculum of 250 pre-J2s for soybean hairy roots, as compared with 500 for whole roots, as the former tissue is much smaller/thinner than the latter. Each replicate consisted of 10 healthy root tips [white and with strong green fluorescent protein (GFP) fluorescence] transferred from a maintenance plate onto solid medium in a 150 mm×25 mm Petri dish. Root tips were inoculated 2–3 d after transfer. The number of J4/adult females was counted with a stereo microscope at 28 dpi. Statistical comparisons were made with the *t*-test in JMP Pro 11.

### GUS histochemical staining

Transgenic hairy roots were inoculated with surface-sterilized *H. glycines* in 6-well plates (250 pre-J2s per well) similar to as described in Baum *et al.* (2000). Infected and uninfected roots were removed from the solid medium, the solid medium was removed, and then roots were placed back into the empty 6-well plates and subjected to histochemical staining for GUS according to Vitha *et al.* (1995). Substrate solution was vacuum infiltrated into the roots, and then roots were incubated at 37 °C for 1–4 h depending on the construct and efficiency of infiltration. All roots for all constructs (except EV control; always 4 h) were incubated in substrate until they reached maximum staining intensity, and before background was observed. Thus, end point staining intensity was purposely comparable for all constructs (except EV control; no staining), but the presence/absence of specific staining was what varied between promoter–GUS constructs. For the syncytium formation phase (J2 and J3 syncytia), infected roots were stained at 5 and 8 dpi. For the syncytium maintenance phase (J4/adult female syncytia), infected roots were stained at 15 dpi. Stained roots were mounted in ddH_2_O or glycerol and observed with a stereo microscope. Images were taken with an AxioCam HR 13 Megapixel Camera (Zeiss). Since there was no GUS staining in EV control roots, any specific staining in the tissue of interest was considered to be positive.

To measure promoter activity, only the healthy roots that grew inside the solid medium (white and with strong GFP fluorescence), as opposed to some root tips that can tend to grow upwards outside of the medium and dry out, were collected and stained. Thus, all of the roots included had the potential for staining. Many of these healthy roots were imaged. For uninfected roots, the percentage of *n*=20 of the healthy roots stained and imaged was scored for the presence or absence of GUS staining and averaged over three different experiments. For infected roots, the percentage of *n*=20 ‘healthy’ roots each with a particular *H. glycines* life stage with an observable syncytium was scored for the presence or absence of GUS staining and averaged over three different experiments. Multiple statistical comparisons were made by ANOVA and Tukey–Kramer HSD post-hoc test in JMP Pro 11.

## Results

### *MIR396* and *GRF* gene families are active in young soybean roots

To begin, we first needed to identify the *MIR396* and *GRF* gene families, and then determine if these genes are expressed in soybean roots. Eleven soybean pre-miR396 sequences were found in miRBase (pre-miR396a–k), and unique genomic co-ordinates were identified for all but pre-miR396d and g at Soybase (Schmutz *et al.*, 2010) (Supplementary Table S2). Pre-miR396d and g are at the same genomic location and, thus, the latter was removed from our study. Modeled *in silico* stem–loop structures for all but pre-miR396h formed an miR396/miR396* duplex within the stems (Supplementary Fig. S1A). Thus, there are nine canonical *MIR396* genes. Also, phylogenetic analysis (to inform primer design for expression analyses) identified four subfamilies (Supplementary Fig. S1B: subfamily 1, pre-miR396a/i; subfamily 2, pre-miR396e/h/j; subfamily 3, pre-miR396c/f; subfamily 4, pre-miR396b/d/k). Pre-miR396d and k within subfamily 4 are identical. Hence, there are eight unique pre-miR396 sequences. Moreover, pre-miR396 sequences within all four subfamilies were almost identical, with only a few nucleotide mismatches within the loops. Furthermore, three mature miR396 molecules were found to be produced by the eight unique pre-miR396, differentiated by the 3'-most nucleotide(s). Finally, qRT–PCR on RNA from roots of 10-day-old soybean cv. Williams 82 seedlings detected expression, albeit variable, of all pre-miR396 subfamilies and individual miR396 molecules (Supplementary Fig. S2A, B). Thus, there are eight unique, canonical pre-miR396 and three mature miR396 molecules, of which all four pre-miR396 subfamilies and mature miR396 molecules are expressed in young soybean roots.

Putative miR396 target sites were identified in all but one *GRF* mRNA sequence (*GRF25*; Supplementary Fig. S3). Thus, *GRF1–24* have the potential for post-transcriptional regulation by miR396, consistent with the findings of Liu *et al.* (2017). *GRF2* and *7* mRNAs were undetectable in 10-day-old soybean roots by qRT–PCR using as many as 40 cycles, while the remaining *GRF* mRNAs were detected, albeit with variable expression levels (Supplementary Fig. S2C). Also, *GRF5/24*, *9*, *14*, *18*, *20–23*, and *25* mRNAs resulted in much greater expression levels compared with the other 13 *GRF* genes. Thus, 23 *GRF* genes are active with varying levels of expression in young soybean roots, consistent with the expression of the *MIR396* gene family.

### Eleven *GRF* genes are up-regulated during the *H. glycines* syncytium formation phase

**Fig. 1.**
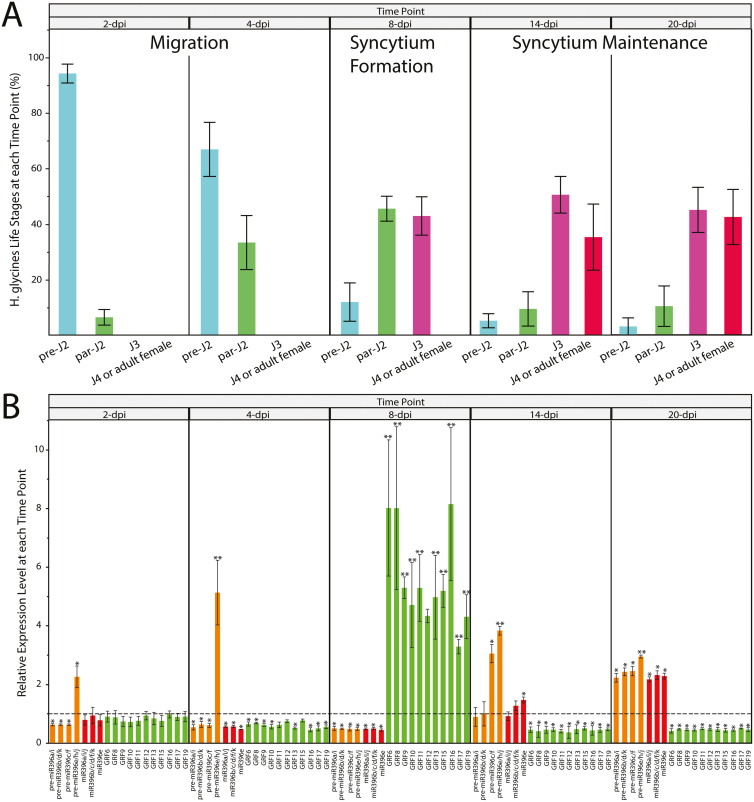
Time course expression analysis of the miR396–*GRF6/8–13/15–17/19* regulatory network during *H. glycines* infection. (A) Assessment of syncytial phases in *H. glycines*-infected soybean roots (*n*=5 plants). At 2 dpi, an average of 94.1% of *H. glycines* were still in the migratory pre-parasitic (pre)-J2 stage while the few remaining were par-J2s. At 4 dpi, an average of 66.8% of *H. glycines* were in the pre-J2 stage and the remainder were par-J2s. Thus, we determined 2 and 4 dpi as early and late migration, respectively. By 8 dpi, an average of 45.4% and 42.8% of *H. glycines* were in par-J2 or early J3 stages, respectively, while the remaining few were pre-J2s. Thus, 8 dpi was designated as the syncytium formation phase. By 14 and 20 dpi, the majority of *H. glycines* were in late J3, J4, or adult female stages and, thus, these time points were designated as the syncytium maintenance phase. (B) Time course qRT–PCR analysis of pre-miR396 subfamilies, miR396 molecules, and the 11 *GRF* genes. Expression levels are relative to mock-inoculated; baseline expression is set to 1.0 and indicated with a dashed line. *P<0.05; **P<0.01. (A, B) Error bars represent ±1 SD from the mean.

We next examined whether *GRF* genes change expression in response to *H. glycines* infection, specifically during the syncytium formation phase. However, since we always observe extensive variability in *H. glycines* life stages within each individual soybean root system, especially between 2 and 21 dpi, we first had to determine which time point cumulatively corresponded to the syncytium formation phase in our infection system. For this analysis, we inoculated soybean roots with *H. glycines* and, at 2, 4, 8, 14, and 20 dpi, evaluated in which life stage the majority of *H. glycines* juveniles were (Fig. 1A). Cumulatively, we determined <2 to 4 dpi as the migration phase, 5–13 dpi as the peak syncytium formation phase, and 14 to >20 dpi as the syncytium maintenance phase in our infection assay. Although the cumulative syncytium formation phase appears somewhat delayed compared with the findings of Ithal *et al.* (2007*b*), which could be due to both biological and technical differences, we did observe syncytia being formed as early as 2 dpi (5.9% par-J2), and more at 4 dpi (33.2% par-J2). However, cumulatively, the large majority of syncytium formation was occurring during the 8 dpi time point, with nearly 90% of *H. glycines* in either par-J2 or J3 stages. By 14 dpi, nearly 90% of *H. glycines* were in J3 or J4/adult female stages, indicating syncytium maintenance.

A qRT–PCR screen was performed on RNA isolated from the 8 dpi roots for all 25 *GRF* genes. Interestingly, 11 *GRF* mRNAs resulted in significantly increased expression compared with mock-inoculated roots (between 3- and 8.5-fold increases, *P*<0.01), while 12 *GRF* mRNAs were unchanged and *GRF2* and *7* remained undetected (Supplementary Fig. S4). The 11 up-regulated *GRF* genes were *GRF6*, *8*, *9–13*, *15*–*17*, and *19*. These results suggested that a network of 11 different *GRF* genes are involved in *H. glycines* infection.

### The miR396–*GRF6/8–13/15–17/19* regulatory network delineates the phases of the *H. glycines* syncytium

Having determined that the *MIR396* gene family members are transcriptionally active in roots and that 11 *GRF* genes are up-regulated in response to *H. glycines* during the cumulative syncytium formation phase, it was of interest to examine the anticipated post-transcriptional silencing of these *GRF* genes by miR396 during various stages of infection. We used qRT–PCR to quantify the expression of the four pre-miR396 subfamilies, the three miR396 molecules, and the 11 *GRF* genes, at 2, 4, 8, 14, and 20 dpi, relative to mock-inoculated roots (Fig. 1B). With the exception of pre-miR396e/h/j, all pre-miR396 and miR396 as well as the *GRF* genes showed no significant changes or only a slight down-regulation during the cumulative migration time points (2–4 dpi). Interestingly, pre-miR396e/h/j showed significant up-regulation during the migration time points, but this up-regulation was not reflected by increased expression of the miR396 molecules, a possible indication of impaired miRNA maturation processing.

Strikingly, during the syncytium formation phase at 8 dpi, all pre-miR396 subfamilies and miR396 molecules showed significant down-regulation of >2-fold, and this down-regulation was accompanied by significant up-regulation of *GRF* genes showing between 3- and 8.5-fold mRNA increases (Fig. 1B). Furthermore, at 14 dpi (i.e. the switch to syncytium maintenance), pre-miR396c/f (subfamily 3) and pre-miR396e/h/j (subfamily 2) were significantly up-regulated between 3- and 4-fold, respectively, while pre-miR396a/i (subfamily 1) and pre-miR396 b/d/k (subfamily 4) were no longer down-regulated. Moreover, miR396e was significantly up-regulated >1.5-fold, while miR396a/i/j and miR396b/c/d/f/k were no longer down-regulated. Conversely, all 11 *GRF* genes were significantly down-regulated >2-fold. Furthermore, at 20 dpi, all pre-miR396 subfamilies and miR396 molecules were significantly up-regulated between 2.5- and 3.5-fold, while all 11 *GRF* genes remained significantly down-regulated >2-fold. The opposite expression patterns of miR396 and *GRF* genes pointed to post-transcriptional silencing of the 11 *GRF* genes by miR396 during *H. glycines* infection. These results indicated that the miR396–*GRF6/8–13/15–17/19* regulatory network delineates the formation and maintenance phases of the *H. glycines* syncytium.

### *GRF6*, *8*, *9–13*, *15–17,* and *19* are post-transcriptionally regulated by miR396 during *H. glycines* infection

**Fig. 2.**
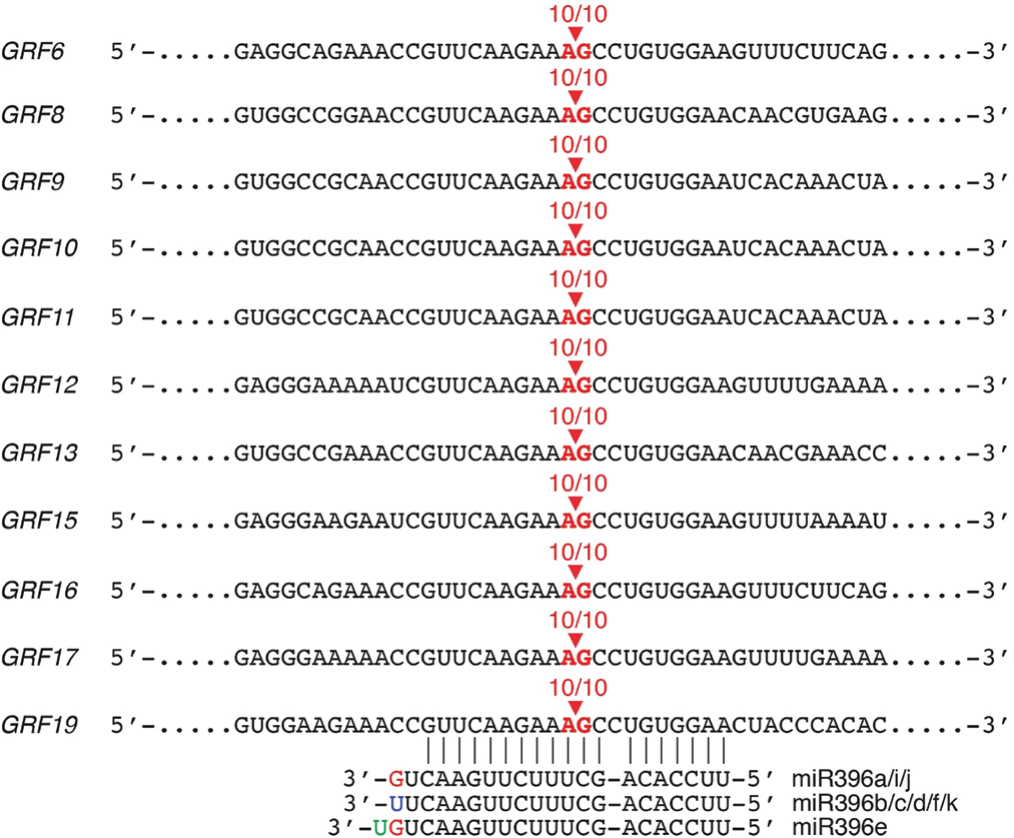
miRNA cleavage assays for *GRF6*, *8–13*, *15–17*, and *19*. Ten different clones for each *GRF* degradation product were analyzed by DNA sequencing. The number of clones that resulted in the indicated cleavage positions within the miR396 target sites is indicated.

To determine whether the gene expression changes of the 11 *H. glycines*-responsive *GRF* genes are the results of their post-transcriptional regulation by miR396 during infection, we performed a 5' RLM-RACE assay on the 14 dpi RNA (i.e. during syncytium maintenance, the time point when down-regulated; see Fig. 1). Cloning and sequencing of the RLM-RACE clones indicated that the cleavage of all 11 *GRF* transcripts occurred within their miR396 target sites between positions 10 and 11 (Fig. 2, 10/10 clones). These results are consistent with previous reports for Arabidopsis *GRF* genes (Jones-Rhoades and Bartel, 2004; Hewezi *et al.*, 2012) and confirmed that *GRF* mRNAs are post-transcriptionally regulated by miR396 in *H. glycines*-infected soybean roots, during the cumulative syncytium maintenance phase.

**Fig. 3.**
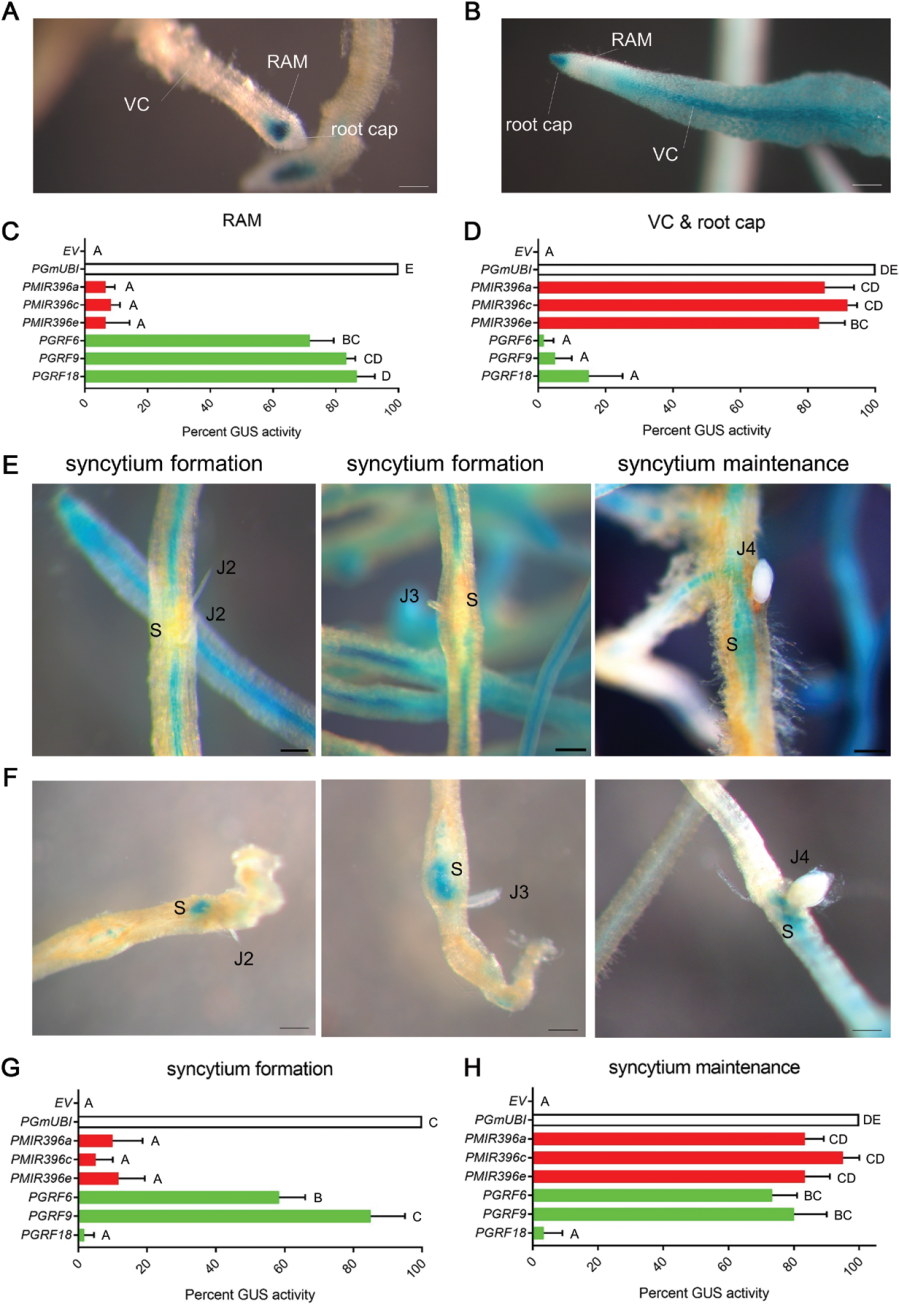
GUS histochemical analyses for selected *MIR396* and *GRF* promoters in uninfected and *H. glycines*-infected soybean roots. (A) Representative image for the native activity of *GRF6*, *GRF9,* and *GRF18* promoters (shown is *GRF9*). (B) Representative image for the native activity of *MIR396a*, *MIR396c*, and *MIR396e* promoters (shown is *MIR396c*). (C) Percentage of GUS-stained roots (*n*=20 roots) in the root apical meristem (RAM) for *MIR396a*, *MIR396c*, *MIR396e*, *GRF6*, *GRF9*, and *GRF18* promoters, with empty vector (EV) and constitutive *PGmUBI* included as negative and positive controls, respectively. (D) Percentage of GUS-stained roots (*n*=20 roots) in the vascular cylinder (VC) and root cap for *MIR396a*, *MIR396c*, *MIR396e*, *GRF6*, *GRF9*, and *GRF18* promoters, with EV and constitutive *PGmUBI* included as negative and positive controls, respectively. (E) Representative images for the activity of *MIR396a*, *MIR396c*, and *MIR396e* promoters within the syncytium during formation (5 and 8 dpi) and maintenance (15 dpi) phases (shown is *MIR396c*). (F) Representative images for the activity of *GRF6*, *GRF9*, and *GRF18* promoters within the syncytium during formation and maintenance phases (shown is *GRF9*). (G) Percentage of GUS-stained roots (*n*=20 infected roots) in the syncytium during the formation phase for *MIR396a*, *MIR396c*, *MIR396e*, *GRF6*, *GRF9*, and *GRF18* promoters, with EV and constitutive *PGmUBI* included as negative and positive controls, respectively. (H) Percentage of GUS-stained roots (*n*=20 infected roots) in the syncytium during the maintenance phase as in (G). (A, B, E, F) Scale bars=0.5 mm. (C, D, G, H) Shown is the mean percentage GUS activity from three independent experiments. Error bars represent ±1 SD from the mean.

Then, we examined whether *GRF* genes may be post-transcriptionally regulated specifically in syncytia. We examined the spatiotemporal expression patterns of *GRF6* and *GRF9*, which were two of the most highly up-regulated *GRF* genes during the cumulative syncytium formation phase (Fig. 1B), as well as *GRF18* as a control that is highly expressed in roots (Supplementary Fig. S2C), but unresponsive to *H. glycines* infection (Supplementary Fig. S4), and *MIR396a*, *MIR396c*, and *MIR396e* to represent all miR396 molecules (see Supplementary Fig. S1A). We generated transgenic hairy roots expressing promoter:GUS fusion constructs for all six genes, and included EV and the constitutive soybean *polyubiqutin* promoter (*PGmUBI*; Hernandez-Garcia *et al.*, 2009) as negative and positive controls, respectively. The histochemical localization of GUS activity directed by these promoters was assayed under both uninfected and *H. glycines*-infected conditions. Under uninfected conditions, *PGRF6*, *PGRF9*, and *PGRF18* produced strong GUS staining within the root apical meristem (RAM) with minimal activity in the vascular cylinder and root cap (Fig. 3A, C, D). *PMIR396a*, *PMIR396c*, and *PMIR396e* produced consistent GUS activity in the vascular cylinder and in the root cap, but were mostly absent from the RAM (Fig. 3B, C, D). As controls, no GUS staining was observed in any EV roots, and all *PGmUBI:GUS* roots were strongly stained in all tissues (Fig. 3C, D).

Under *H. glycines*-infected conditions, *PMIR396a*, *PMIR396c*, and *PMIR396e* showed clear down-regulation in the syncytia induced by J2 and early J3 nematodes (syncytium formation phase), but became very active in the syncytia of J4 nematodes (syncytium maintenance phase) (Fig. 3E, G, H). In contrast, *PGRF6* and, in particular, *PGRF9* showed sustained, high activation in the syncytia induced by J2, J3, and J4 nematodes (Fig. 3F–H). Interestingly, the strong activity of *PGRF9* was observed in a significantly greater percentage of J2 and early J3 syncytia compared with *PGRF6* (Fig. 3G), but this difference was no longer significant in syncytia of J4/adult females (Fig. 3H). Also, and as expected, consistent with the mRNA level (Supplementary Fig. S4), *PGRF18* showed essentially no activity in syncytia of any *H. glycines* life stage (Fig. 3G, H). It is noteworthy that *MIR396* and *GRF* promoter activities outside of syncytia at all time points evaluated were unchanged, closely mirroring the uninfected condition (Supplementary Fig. S5), strongly supporting that the changes in mRNA in response to *H. glycines* infection (Fig. 1B) occurred specifically in syncytia. Furthermore, as controls, and as in uninfected roots, no GUS staining was observed anywhere in any *H. glycines*-infected EV roots, and all *H*. *glycines*-infected *PGmUBI:GUS* roots were strongly stained in all tissues, including syncytia (Fig. 3G, H). Moreover, the strong activities of these three *MIR396*, *GRF6*, and *GRF9* promoters in syncytia during the maintenance phase, in combination with the identified mRNA degradation products (Fig. 2), strongly support that the concomitant down-regulation of the respective *GRF* mRNAs (Fig. 1B) occurs post-transcriptionally from the miR396 expression spike.

### Overexpression of pre-miR396 in soybean roots substantially reduces *H. glycines* development to adult females

**Fig. 4.**
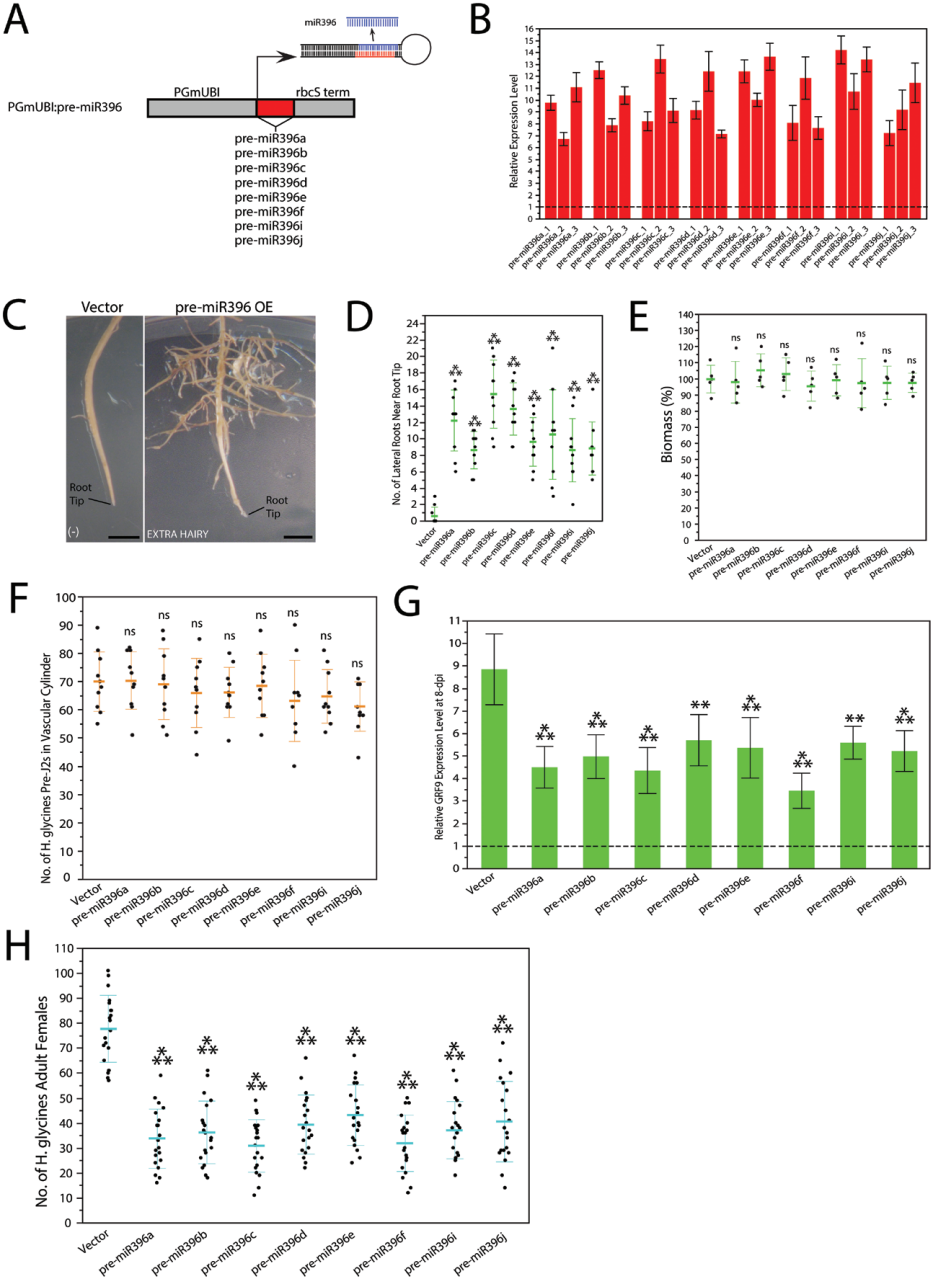
Overexpression of pre-miR396 in soybean roots. (A) Overexpression constructs. (B) qRT–PCR analysis on transgenic pre-miR396-overexpressing roots; three events per construct. Expression levels are relative to empty vector control; baseline expression is set to 1.0 and indicated with a dashed line. (C) *EXTRA HAIRY* phenotype caused by pre-miR396 overexpression. Scale bars=0.5 cm. (D) Comparisons between the number of lateral roots within 2.5 cm from the root tips for pre-miR396-overexpressing and empty vector control roots (*n*=10). (E) Comparisons between biomasses of pre-miR396-overexpressing and empty vector control roots (*n*=5) 1 week after transfer to new maintenance plates. Biomasses were measured as the percentage of dry root weight compared with empty vector control; empty vector control mean was set to 100%. (D, E) Data are representative of three independent experiments. (F) Comparisons between the number of *H. glycines* pre-J2s within the vascular cylinder of pre-miR396-overexpressing and empty vector control roots (*n*=10). (G) qRT–PCR analysis of *GRF9* in *H. glycines*-infected pre-miR396-overexpressing and empty vector control roots at 8 dpi. Expression levels are relative to mock-inoculated roots for each construct; baseline expression is set to 1.0 and indicated with a dashed line. (H) Comparisons between the number of *H. glycines* adult females that developed on pre-miR396-overexpressing and empty vector control roots (*n*=20). (F, H) Data are representative of two independent experiments. (D–H) Error bars represent ±1 SD from the mean. **P<0.01; ***P<0.001; ns, not significant; all are statistically compared with empty vector control.

All eight unique, canonical pre-miR396 were overexpressed in transgenic soybean hairy roots to determine whether interfering with the miR396–*GRF6/8–13/15–17/19* regulatory network would modify susceptibility to *H. glycines*. *PGmUBI* was used for overexpression (Fig. 4A). Transgenic soybean cv. Williams 82 roots with high pre-miR396 overexpression (Fig. 4B) were selected for phenotyping. We used the same qRT–PCR primers as above to quantify the overexpression as this strategy gave optimal melting curves (as described in the Materials and methods).

All pre-miR396-overexpressing roots resulted in an *EXTRA HAIRY* phenotype (Fig. 4C) characterized by more lateral roots within the first 2.5 cm from the root tips compared with the EV control (Fig. 4D). On the other hand, the overall biomasses (i.e. amount of root tissue generated) for pre-miR396-overexpressing roots were statistically similar to those of the EV control (Fig. 4E) and there were no differences in the number of penetrating *H. glycines* J2s that infected them at 4 dpi (Fig. 4F). In other words, the increased number of lateral roots near the root tips in the pre-miR396-overexpressing roots compared with EV control did not have any effect on the number of J2s that entered the vascular cylinder to initiate formation of syncytia.

At 8 dpi, all pre-miR396-overexpressing roots infected with *H. glycines* resulted in significantly reduced inductions of *GRF9* during the syncytium formation phase compared with EV control (Fig. 4G). We selected *GRF9* as a marker due to its particularly high promoter activity in the syncytium (Fig. 3F–H). Remarkably, in spite of the same number of J2s being inside the roots of pre-miR396 and EV control at 4 dpi (Fig. 4F), all pre-miR396-overexpressing roots resulted in highly significant reductions in the number of *H. glycines* adult females that developed by 28 dpi (Fig. 4H). Thus, overexpression of all canonical pre-miR396 substantially reduces susceptibility to *H. glycines* not by affecting the number of J2s that enter the vascular cylinder to initiate syncytia, but by inhibiting the development of the nematodes to the adult female stage, in association with silencing of *GRF* expression during the syncytium formation phase.

### RNAi of *GRF9* phenocopies pre-miR396 overexpression

**Fig. 5.**
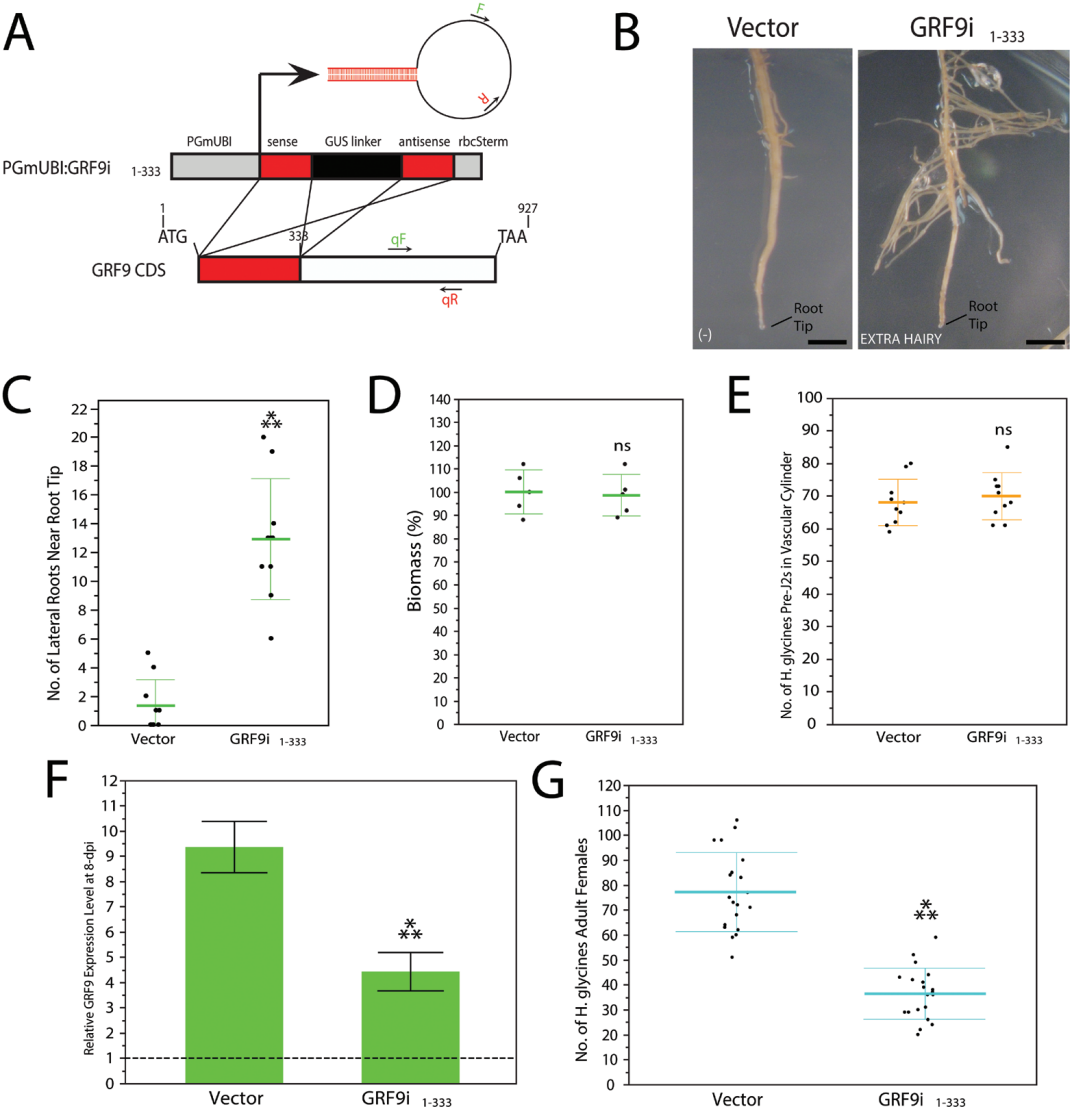
RNAi of *GRF9* in soybean roots. (A) RNAi construct. Annealing sites for the primers used for RT–PCR diagnosis of transgene expression (F and R), and qRT–PCR analysis of *GRF9* (qF and qR) are indicated. (B, C) *EXTRA HAIRY* phenotype for *GRF9i*_*1–333*_, as in Fig. 4C and D). (D) Comparison between biomasses of *GRF9i*_*1–333*_ and empty vector control roots, as in Fig. 4E. (E) Comparisons between the number of *H. glycines* pre-J2s within the vascular cylinder of *GRF9i*_*1–333*_ and empty vector control roots, as in Fig. 4F. (F) Comparison between *GRF9* relative expression levels in *H. glycines*-infected *GRF9i*_*1–333*_ and empty vector control roots at 8 dpi, as in Fig. 4G. (G) Comparisons between the number of *H. glycines* adult females that developed on *GRF9i*_*1–333*_ and empty vector control roots, as in Fig. 4H.

Due to *GRF9* being among the most highly up-regulated *GRF* genes during the cumulative syncytium formation phase (Fig. 1B), having by far the highest steady-state mRNA levels among the 11 *H. glycines* responsive *GRF* genes (Supplementary Fig. S2C), and its particularly high promoter activity in syncytia (Fig. 3F–H), we next examined the effect of knocking down *GRF9*. An RNAi hairpin construct was generated for *GRF9* and placed under the transcriptional control of the *GmUBI* promoter (Fig. 5A). Transgenic events that were determined to express *GRF9i*_*1–333*_ via RT–PCR were selected for phenotyping.

Interestingly, and, at first, somewhat surprisingly, all *GRF9i*_*1–333*_ soybean roots phenocopied the *EXTRA HAIRY* phenotype that was observed for pre-miR396-overexpressing roots (Fig. 5B) resulting in similar numbers of lateral roots within the first 2.5 cm from the root tip (Fig. 5C). Also, as observed for pre-miR396-overexpressing roots, *GRF9i*_*1–333*_ roots showed no difference in root biomass (Fig. 5D) or in the number of penetrating *H. glycines* J2s that infected them at 4 dpi compared with EV control (Fig. 5E).

As expected, induction of *GRF9* during the syncytium formation phase at 8 dpi was significantly reduced in the *GRF9i*_*1–333*_ roots (Fig. 5F) to a similar level to that at pre-miR396 overexpression. However, *GRF9i*_*1–333*_ also resulted in moderately reduced induction of the other 10 *H. glycines*-responsive *GRF* genes at 8 dpi, of which the *GRF* genes most similar to *GRF9* (*GRF10*, *GRF11*,and *GRF12*; Supplementary Fig. S6) were significantly reduced (Supplementary Fig. S7). On the other hand, induction of *GRF18* at 8 dpi was unaffected in *GRF9i*_*1–333*_ roots as it is unresponsive to *H. glycines* infection (Supplementary Fig. S7) and, thus, all of the silencing effects with *GRF9i*_*1–333*_ during infection are likely to be limited to the 11 *H. glycines*-responsive *GRF* genes, and *GRF9* in particular. Strikingly, similar to pre-miR396 overexpression, *GRF9i*_*1–333*_ roots resulted in a highly significant reduction in the number of *H. glycines* that developed to adult females (Fig. 5G). Thus, RNAi-mediated down-regulation of *GRF9*, and to a moderate extent the other 10 *H. glycines*-responsive *GRF* genes, phenocopied the reduced susceptibility phenotypes caused by pre-miR396 overexpression. These results underscore the necessity of adequate expression of these 11 *GRF* genes, and in particular *GRF9*, during the syncytium formation phase for productive *H. glycines* infections.

### Overexpression of miR396-resistant *GRF9* resembles pre-miR396 overexpression and *GRF9* RNAi

**Fig. 6.**
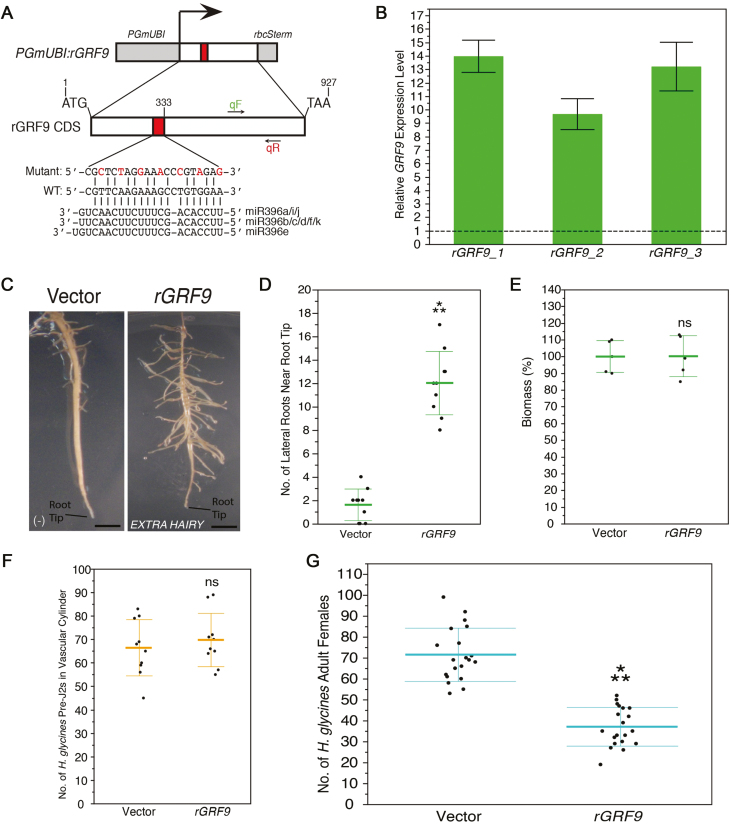
Overexpression of an miR396-resistant mutant of *GRF9* in soybean roots. (A) Overexpression construct for miR396-resistant *GRF9* (*rGRF9*). An illustration of the synonymous mutations introduced into the miR396 target site is shown. Annealing sites for the primers used for qRT–PCR analysis of *GRF9* levels are shown. (B) qRT–PCR analysis of *GRF9* in transgenic *rGRF9*-overexpressing roots. Expression levels are relative to empty vector control; baseline expression is set to 1.0 and indicated with a dashed line. (C, D) *EXTRA HAIRY* developmental phenotype for *rGRF9* overexpression, as in Fig. 4C and D. (E) Comparison between biomasses of *rGRF9*-overexpressing and empty vector control roots, as in Fig. 4E. (F) Comparisons between the number of *H. glycines* pre-J2s within the vascular cylinder of *rGRF9*-overexpressing and empty vector control roots, as in Fig. 4F. (G) Comparisons between the number of *H. glycines* adult females that developed on *rGRF9*-overexpressing and empty vector control roots, as in Fig. 4H.

Finally, to demonstrate the necessary homeostasis in the miR396–*GRF6/8–13/15–17/19* regulatory network during *H. glycines* infection, as observed in the Arabidopsis–*H. schachtii* model (Hewezi *et al.*, 2012), we overexpressed a synonymous miR396-resistant (*r*)*GRF9* mutant, under *PGmUBI* transcriptional control (Fig. 6A). *GRF9* was selected for reasons explained above. Transgenic soybean roots determined to overexpress *rGRF9* at high levels via qRT–PCR were selected for phenotyping (Fig. 6B).

Comparably with results from Rodriguez *et al.* (2015) and Hewezi *et al.* (2012) in Arabidopsis, *rGRF9*-overexpressing roots also showed the *EXTRA HAIRY* phenotype observed for pre-miR396 overexpression and *GRF9i*_*1–333*_ roots (Fig. 6C, D). Again, this manipulation did not alter the overall root biomass (Fig. 6E) or the number of penetrating *H. glycines* J2s that entered the vascular cylinder to initiate syncytium formation compared with EV control (Fig. 6F). However, overexpression of *rGRF9* in soybean roots resulted in a highly significant reduction in the number of *H. glycines* that developed to adult females compared with EV control (Fig. 6G). As an additional control, in a separate experiment, overexpression of *GUSPlus* had no effect on the number of adult females (Supplementary Fig. S8). Thus, overexpression of *rGRF9* highly resembled the reduced susceptibility phenotype caused by pre-miR396 overexpression and RNAi knockdown of *GRF9* (and, to a moderate extent, the other *H. glycines*-responsive *GRF* gnes). Collectively, these *in vivo* studies indicated that homeostasis in the miR396–*GRF6/8–13/15–17/19* regulatory network is essential for productive *H. glycines* infections.

## Discussion

Previously, the miR396–*GRF1/3* regulatory module was shown to be a master regulator of syncytium development in the model cyst nematode interaction between *H. schachtii* and Arabidopsis (Hewezi *et al.*, 2012). Here, we investigated whether this syncytium post-transcriptional regulatory system is conserved in the interaction between *H. glycines* and soybean, which is of interest for at least two reasons: (i) for the obvious, potential translational benefit; and (ii) no study has addressed whether findings made in this model pathosystem are applicable to the *H. glycines*–soybean pathosystem. We have found that a network involving nine canonical *MIR396* genes (eight unique) and, specifically, 11 *GRF* genes delineates the *H. glycines* syncytium formation and maintenance phases in soybean via transcriptional regulation of both *MIR396* and *GRF* genes, and post-transcriptional regulation of *GRF* genes by miR396. Disrupting the balance in this network either by reducing *GRF* expression during the syncytium formation phase (either by pre-miR396 overexpression or RNAi knockdown) or by overexpression of *rGRF9* dramatically inhibits the number of *H. glycines* that develop to adult females. Thus, although involving many more genes in soybean, the miR396–*GRF* regulatory network is clearly conserved between *H. schachtii*–Arabidopsis and *H. glycines*–soybean pathosystems, and a balanced network is essential for productive *H. glycines* infections.

Overexpression of all canonical pre-miR396 in soybean roots resulted in an *EXTRA HAIRY* phenotype (Fig. 4C, D) that was accompanied by *GRF9* silencing during syncytium formation (Fig. 4G). Liu *et al.* (2017) generated Arabidopsis overexpression lines for all soybean pre-miR396, and all except pre-miR396d, f, and j gave similar mutant phenotypes. We suggest that lack of observed mutant phenotypes for soybean pre-miR396d, f, and j could have been due to the use of a surrogate transgenic system. Also, the number of lateral roots near the root tip, which we classified as an *EXTRA HAIRY* phenotype, was not scored in Liu *et al.* (2017), so it is not certain that soybean pre-miR396d, f, and j do not give this phenotype when overexpressed in Arabidopsis. Furthermore, the conclusion in Liu *et al.* (2017) that these soybean pre-miR396 do not function in the plant is not clear since they were capable of cleaving the consensus *GRF* target site in Arabidopsis protoplasts. In the present study, we have provided strong experimental evidence that these soybean pre-miR396 are functional in soybean roots.

Twenty-three *GRF* genes showed varying degrees of expression in young soybean roots, while only two *GRF* genes were undetected (Supplementary Fig. S2C). We compared these data with RNA sequencing (RNA-seq) data that are available for soybean root tips at the soybean functional genomics database (SFGD) (Yu *et al.*, 2014). Even though these RNA-seq data were obtained from root tips as opposed to whole roots, and the plants were grown under different conditions from those in our experiments, we found that our qRT–PCR data were well correlated with the SFGD RNA-seq data (Supplementary Fig. S9; *R*^2^=0.48, *P*<0.001). Thus, this observation validates the accuracy of both our qRT–PCR data for *GRF* expression in whole soybean roots and the SFGD RNA-seq data for root tips. Also, *MIR396* gene family members exhibited varying degrees of expression in young soybean roots (Supplementary Fig. S2A, B), which is consistent with previous findings (Li *et al.*, 2012). However, Li *et al.* (2012) did not mention changes in miR396 abundance in response to *H. glycines*, but in their study soybean plants were grown in *H. glycines*-infested soil and they were only evaluated at a single time point long after infection was established. Tian *et al.* (2017) also did not mention changes in miR396 abundance in response to *H. glycines*, but, again, soybean plants were grown in *H. glycines*-infested soil, probably diluting out the phase-specific changes in developing syncytia (see Fig. 1B). We found that all canonical pre-miR396 and miR396 are down-regulated early during the syncytium formation phase, and up-regulated during the maintenance phase (Fig. 1B), mirroring *MIR396* promoter activities in syncytia (Fig. 3E, G, H). Thus, our finding that expression patterns differ drastically at different time points (i.e. during different phases of syncytium development) during *H. glycines* infection probably explains why these previous studies did not mention miR396.

Liu *et al.* (2017) did not detect expression of the pre-miR396c/f subfamily or *GRF* genes *5*, *6*, *9*, *12*, *13*, *16*, *17*, *22,* and *24* (names according to this paper) in soybean roots, yet we detected them with relatively low Ct values in all biological replicates (Supplementary Fig. S2A, C). In fact, pre-miR396c/f was by far the most highly expressed subfamily (Supplementary Fig. S2A). Also, *MIR396c* (along with *MIR396a* and *MIR396e*), *GRF6,* and *GRF9* promoters were clearly active in soybean roots (Fig. 3A–D). Moreover, Li *et al.* (2012) detected expression of all of these *GRF* genes in soybean root tips, which correlates well with our data (Supplementary Fig. S9). We suggest that the different growth conditions, RNA preparations, qRT–PCR parameters, and/or primers may be the cause for lack of detection of expression of these genes in soybean roots by Liu *et al.* (2017).

Eleven soybean *GRF* genes are specifically up-regulated during the cumulative syncytium formation phase (at 8 dpi), which in our infection system was between 5 and 13 dpi (Fig. 1A), while the other *GRF* genes do not change (Supplementary Fig. S4). Moreover, promoter analyses (Fig. 3F–H) indicate that this up-regulation is specific in syncytia during the formation phase as *GRF* promoters are, for the most part, specific to the RAM in uninfected roots, while syncytia are induced in the vascular cylinder. We also did not observe any changes in promoter activities for the respective *MIR396* and *GRF* genes outside of syncytia (Supplementary Fig. S5). Microarray analysis was previously performed on laser capture-microdissected *H. glycines* syncytia at 2, 5, and 10 dpi (Ithal *et al.*, 2007*b*). However, this study did not present any data on *GRF* genes, which is probably due to a number of factors. For instance, the only genes that were analyzed in the latter study were those that first changed in expression at 2 dpi, and then those genes were subsequently analyzed at 5 and 10 dpi. Thus, it is likely that at 2 dpi *GRF* genes are not yet up-regulated to the point of detection, which would be consistent with our results (Fig. 1B), and may explain why *GRF* genes were not mentioned in the latter study. Other microarray analyses were also performed on *H. glycines*-infected, whole soybean roots, but again no data were presented on *GRF* genes (Alkharouf *et al.*, 2006; Ithal *et al.*, 2007*a*). Lack of data presented for *GRF* genes in these studies could have also been due to insufficient representation on the GeneChip (Ithal *et al.*, 2007a, b) or cDNA (Alkharouf *et al.*, 2006) arrays, or possibly a combination of other factors. Also, many other microarray and RNA-seq studies have been performed on *H. glycines*-infected soybean roots, but the changes that are presented in those studies are representative of resistant reactions. It is noteworthy that our qR–PCR data for *GRF* expression changes during *H. glycines* infection (Fig. 1B) and promoter data in syncytia (Fig. 3F–H) are consistent with the previously published microarray, qRT–PCR, and promoter data for *H. schachtii*-infected Arabidopsis roots (Szakasits *et al.*, 2009; Hewezi *et al.*, 2012).

In the RAM, stem cell progeny undergo rapid cell division to ensure that there are enough cells for proper growth, and these rapidly dividing cells are called the transit-amplifying cells (TACs) (Rodriguez *et al.*, 2015). In Arabidopsis roots, miR396 is abundant in the root cap and stem cell niche (SCN) formed by the quiescent center (QC) and adjacent stem cell initials, while *GRF* genes are abundant in TACs. GRFs promote rapid cell cycling within TACs, and miR396-mediated down-regulation of GRFs results in delayed cell cycling (Rodriguez *et al.*, 2015). We found that soybean *MIR396a*, *MIR396c*, and *MIR396e* promoters are active within the root cap (most probably columella cells and the SCN) and the vascular cylinder leading up to the RAM (Fig. 3B–D), and that *GRF6*, *GRF9*, and *GRF18* promoters are predominantly active within the RAM, most probably TACs (Fig. 5A, C, D). Thus, the function of the miR396–*GRF* regulatory network in soybean roots appears to be similar to that of other plant species (Bazin *et al.*, 2013; Rodriguez *et al.*, 2015), although we do not know if the other *MIR396* and *GRF* promoters have the same patterns of activity throughout the root system during development, which was beyond the scope of our study. Importantly, *GRF* genes are up-regulated in the syncytium during the formation phase concomitant with the down-regulation of miR396 (Figs 1B, 3E–H). Conversely, during the syncytium maintenance phase, *GRF* genes are post-transcriptionally down-regulated by the de-repressed miR396 expression (Figs 1B, 3E–H, 4). We also found that all 11 *GRF* mRNAs are cleaved via miR396 during the syncytium maintenance phase (Fig. 2), but that *GRF6* and *GRF9* promoters remain highly active during this time (Fig. 3F–H), indicating post-transcriptional down-regulation by miR396. Hence, soybean *GRF* genes appear to be regulated in the *H. glycines* syncytium by miR396 in parallel to the RAM, suggesting that GRFs might function to maintain rapid cell cycling in the forming syncytium for proper organ development (Engler and Gheysen, 2013). It is noteworthy that *GRF18*, which we found as the most highly expressed *GRF* in young soybean roots (Supplementary Fig. S2C), specifically in the RAM (Fig. 3A, C, D), and does not change during *H. glycines* infection (Supplementary Fig. S4), has no promoter activity in *H. glycines* syncytia (Fig. 3G, H). Thus, the developmental program of developing *H. glycines* syncytia is clearly different from that of the RAM, involving select *GRF* genes.

When plants are under high pathogen stress, resources are devoted towards defense responses, while growth is stunted and development is delayed. This phenomenon is known as the growth–defense trade-off (Huot *et al.*, 2014). GRFs have been implicated in various abiotic and biotic stress conditions (Liu *et al.*, 2008; Y. Li *et al.*, 2010; Hewezi *et al.*, 2012; Kim *et al.*, 2012; Casadevall *et al.*, 2013; Liu *et al.*, 2017), and regulate the expression of a wide range of genes involved in both developmental processes and defense responses (Liu *et al.*, 2014). GRFs are thus hypothesized to co-ordinate the interactions between defense signaling and growth and developmental pathways (Liu *et al.*, 2014). In this context, GRFs could be thought to promote growth by maintaining rapid cell cycles while simultaneously suppressing defense responses. We found that silencing *GRF* genes enhances soybean lateral root formation near the root tips (Figs 4C, D, 5B, C). This phenotype is probably reflected by a decreased elongation zone (Rodriguez *et al.*, 2015), which may also explain the similar biomasses and numbers of *H. glycines* J2 that penetrated the vascular cylinder to initiate syncytia observed between control and mutant roots (Figs 4E, 5D), yet highly reduced susceptibility to *H. glycines* in mutant roots (Figs 4H, 5G), consistent with *GRF* genes promoting developmental processes and suppressing defenses. Interestingly, however, overexpression of *rGRF9* gave an *EXTRA HAIRY* and highly reduced susceptibility phenotype similar to *GRF* silencing (Fig. 6). Thus, although *GRF* genes are required to maintain proper soybean lateral root numbers, and productive *H. glycines* infections, their precise expression levels, fine-tuned by miR396, are also required (probably too rapid and uncontrolled cell cycling in developing syncytia is also problematic), consistent with the Arabidopsis–*H. schachtii* model (Hewezi *et al.*, 2012).

Feedback regulation of miRNAs by their transcription factor targets has been demonstrated in several studies (Gutierrez *et al.*, 2009; Wang *et al.*, 2009; Wu *et al.*, 2009; Marin *et al.*, 2010; Yant *et al.*, 2010; Hewezi and Baum, 2012). Also, overexpression of *rGRF1* and *3* in Arabidopsis not only down-regulates miR396, but also down-regulates other *GRF* genes as well as wild-type *GRF1* and *3*, respectively, in roots (Hewezi and Baum, 2012). Although some of the co-ordination between miR396 and *GRF* genes can be explained through PLETHORA (Rodriguez *et al.*, 2015) and TCP4 (Rodriguez *et al.*, 2010) transcription factors, it is clear that *MIR396* and *GRF* genes are downstream targets that are negatively regulated by *GRF* genes in roots (Hewezi and Baum, 2012). This complex feedback loop ensures a precise transcriptional equilibrium. Thus, pre-miR396 overexpression, RNAi of *GRF9* (and, to a lesser extent, the other 10 *H. glycines*-responsive *GRF* genes; Supplementary Fig. S6), and *rGRF9* overexpression all resulting in comparable *EXTRA HAIRY* and highly reduced susceptibility phenotypes, consistent with the Arabidopsis–*H. schachtii* model (Hewezi *et al.*, 2012), underscores the likely importance of this complex feedback loop to maintain such an equilibrium in soybean. Future studies that analyze the expression changes in miR396 and *GRF* genes in *rGRF9*-overexpressing roots, for example, will provide a more complete picture of the necessary feedback regulations within the miR396–*GRF6/8–13/15–17/19* regulatory network during *H. glycines* infection.

In summary, we have investigated if a miR396–*GRF* regulatory system operates in the agronomically important interaction between *H. glycines* and soybean. Our results demonstrate that the miR396–*GRF6/8–13/15–17/19* regulatory network delineates the phases of the *H. glycines* syncytium and that interfering in the homeostasis of this network inhibits productive *H. glycines* infections (i.e. greatly reduces the number of adult females that develop). As *H. glycines* is the most economically devastating soybean pathogen causing over US$1 billion in yield losses each year (Koenning and Wrather, 2010), control strategies more effective than the conventional measures (Conley *et al.*, 2011) are urgently needed. Thus, by specifically interfering with this network in syncytia using an *H. glycines*-inducible promoter (thereby avoiding changes to the plant outside of syncytia), this network may serve as a potential target to develop soybean plants with novel, synthetic resistance to *H. glycines*.

## Supplementary data

Supplementary data are available at *JXB* online.

Fig. S1. The soybean *MIR396* gene family.

Fig. S2. Native expression of pre-miR396, miR396, and *GRF* genes in young soybean roots.

Fig. S3. Putative miR396 target sites in *GRF1–24*.

Fig. S4. qRT–PCR screen of *GRF* genes in *H. glycines*-infected roots at 8 dpi.

Fig. S5. Activities of selected *MIR396* and *GRF* promoters in *H. glycines*-infected soybean roots outside of syncytia.

Fig. S6. Multiple sequence (MUSCLE) alignment of soybean *GRF* genes.

Fig. S7. qRT–PCR analysis of *H. glycines*-responsive *GRF* genes in *GRF9i*_*1–333*_ roots.

Fig. S8. *PGmUBI*-driven expression of *GUSPlus* does not change susceptibility to *H. glycines* infection.

Fig. S9. Comparison between SFGD root tip RNA-seq data and our whole root qRT–PCR data for steady-state expression of soybean *GRF* genes.

Table S1. Complete list of primers used in our study.

Table S2. Soybase genome co-ordinates and gene calls for *MIR396* and *GRF* genes.

Supplementary Figures S1-S9 and Tables S1-S2
