# Supplementary material for: Homeostasis in the soybean miRNA396–GRF network is essential for productive soybean cyst nematode infections
Source: J Exp Bot. 2019 Jan 30;70(5):1653–68. doi: 10.1093/jxb/erz022 (PMC6411377; doi:10.1093/jxb/erz022)
Supplement: Supplementary Figures S1-S9 and Tables S1-S2 [file erz022_suppl_supplementary_figures_s1-s9_and_tables_s1-s2.pdf]

Homeostasis in the soybean microRNA396-*GRF* network is essential for productive soybean cyst nematode infections

Jason B. Noon[1], Tarek Hewezi[2], Thomas J. Baum[1]*

[1]Department of Plant Pathology and Microbiology, Iowa State University, Ames, Iowa 50011, U.S.A.

[2]Department of Plant Sciences, University of Tennessee, Knoxville, Tennessee 37996, U.S.A.

*Author for correspondence: Thomas J. Baum, phone: +1-515-294-5420, fax: +1-515-294-9420, email: tbaum@iastate.edu

Email (JBN[#]): jbnoon@bu.edu

Email (TH): thewezi@utk.edu

[#] JBN's current affiliation: Pulmonary Center, Boston University School of Medicine, Boston, Massachusetts 02118, U.S.A.

Supplementary Data Files:

Figure S1 – S9

Table S1 – S2

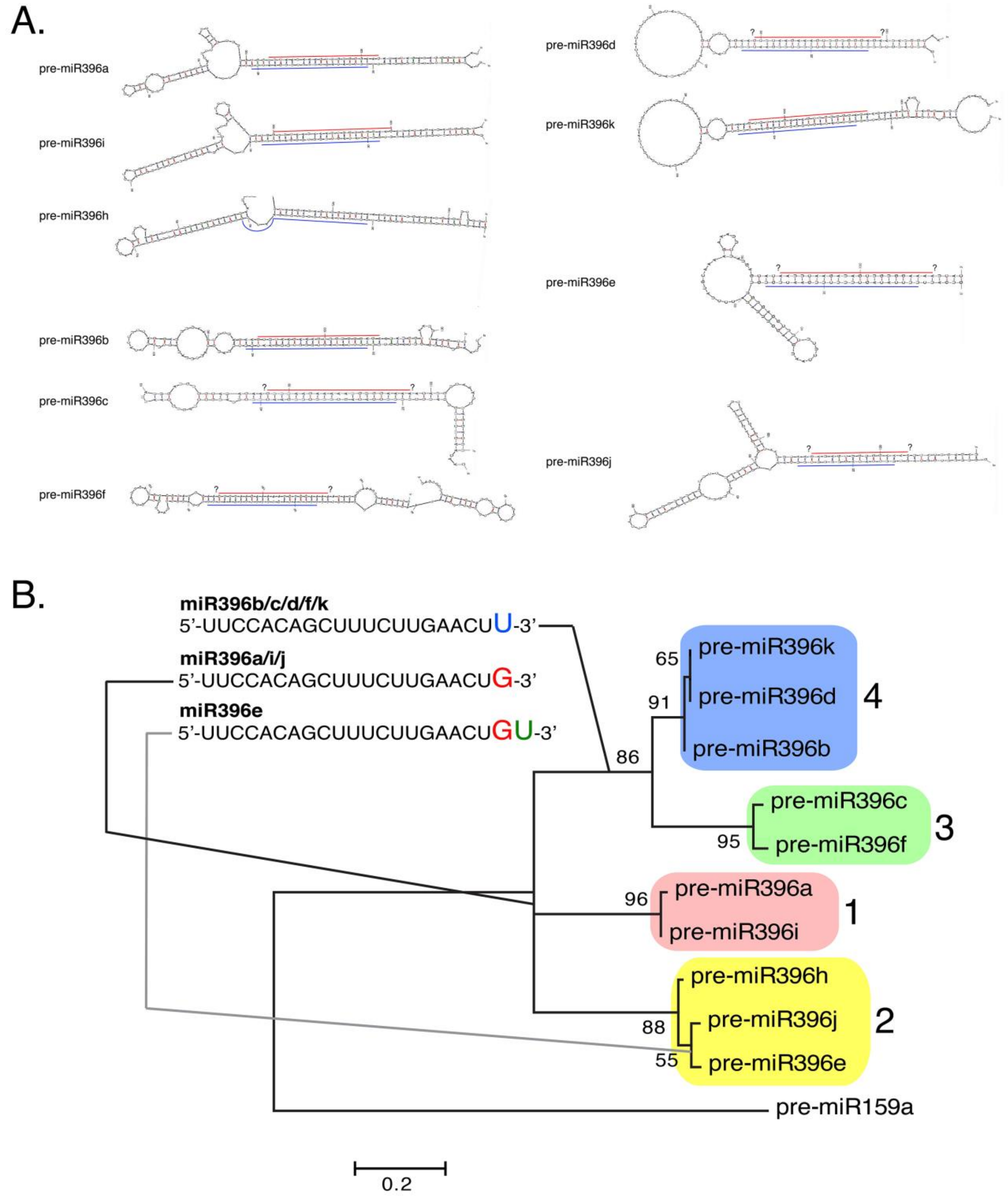

Fig. S1. The soybean *MIR396* gene family. (A) *In silico* pre-miR396 stem-loop structures grouped according to structural similarities. miR396 and miR396* are indicated with blue and red lines, respectively. Question marks on miR396* indicate that the exact 5'- and 3'-ends are

unknown due to lack of available sequence information in miRBase. (B) Maximum Likelihood phylogenetic tree of the soybean *MIR396* gene family. The four well-supported pre-miR396 subfamilies are indicated. The timing of appearance of each miR396 molecule is indicated at the corresponding branch. The line connecting miR396e to the corresponding branch is colored grey to indicate a more recent appearance. Scale bar equals the number of nucleotide substitutions per site.

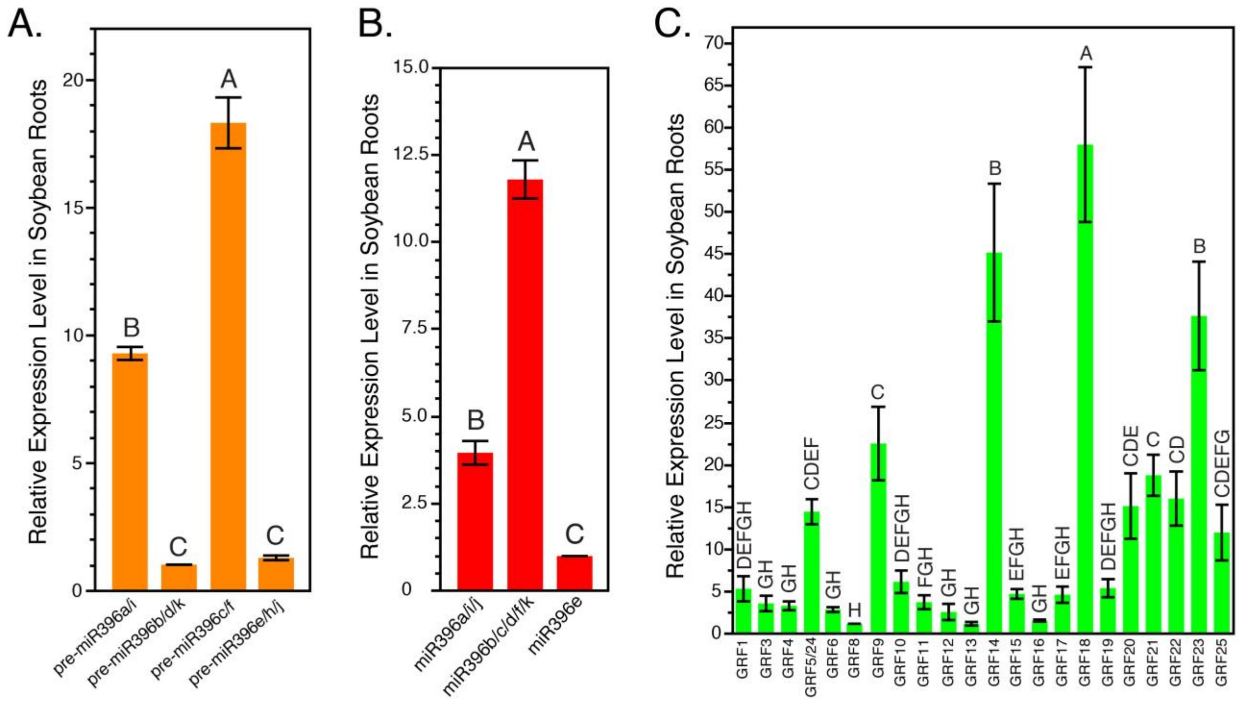

Fig. S2. Native expression of pre-miR396, miR396 and *GRFs* in young soybean roots. (A) qRT-PCR analysis of the four pre-miR396 subfamilies. Expression levels are relative to pre-miR396b/d/k. (B) qRT-PCR analysis of the three mature miR396 molecules. Expression levels are relative to miR396e. (C) qRT-PCR analysis of *GRF1-25*. Expression levels are relative to *GRF8*. (A-C) Error bars represent ± one standard deviation from the mean. Significance groups are shown ($P < 0.05$).

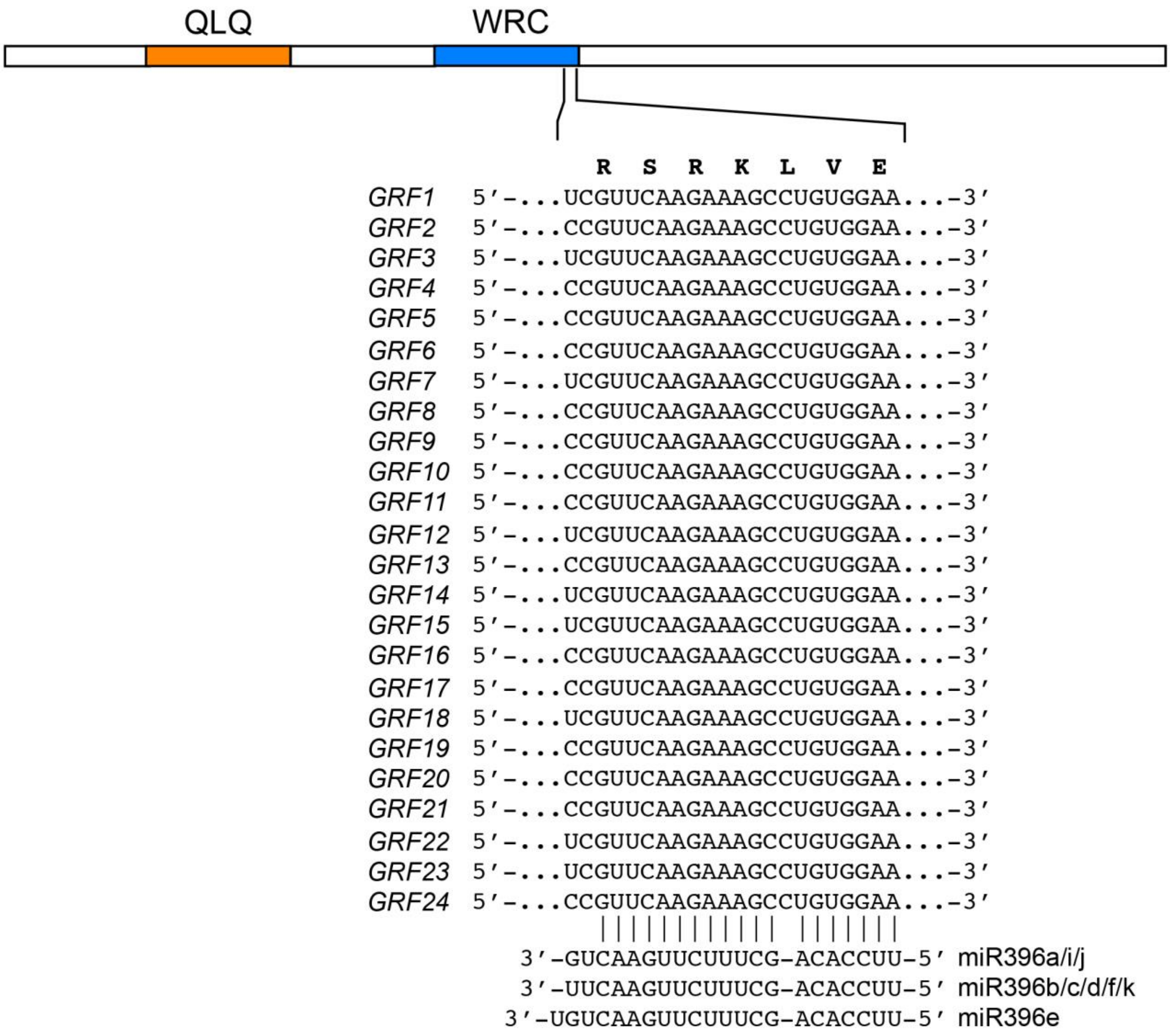

Fig. S3. Putative miR396 target sites in *GRF1-24*.

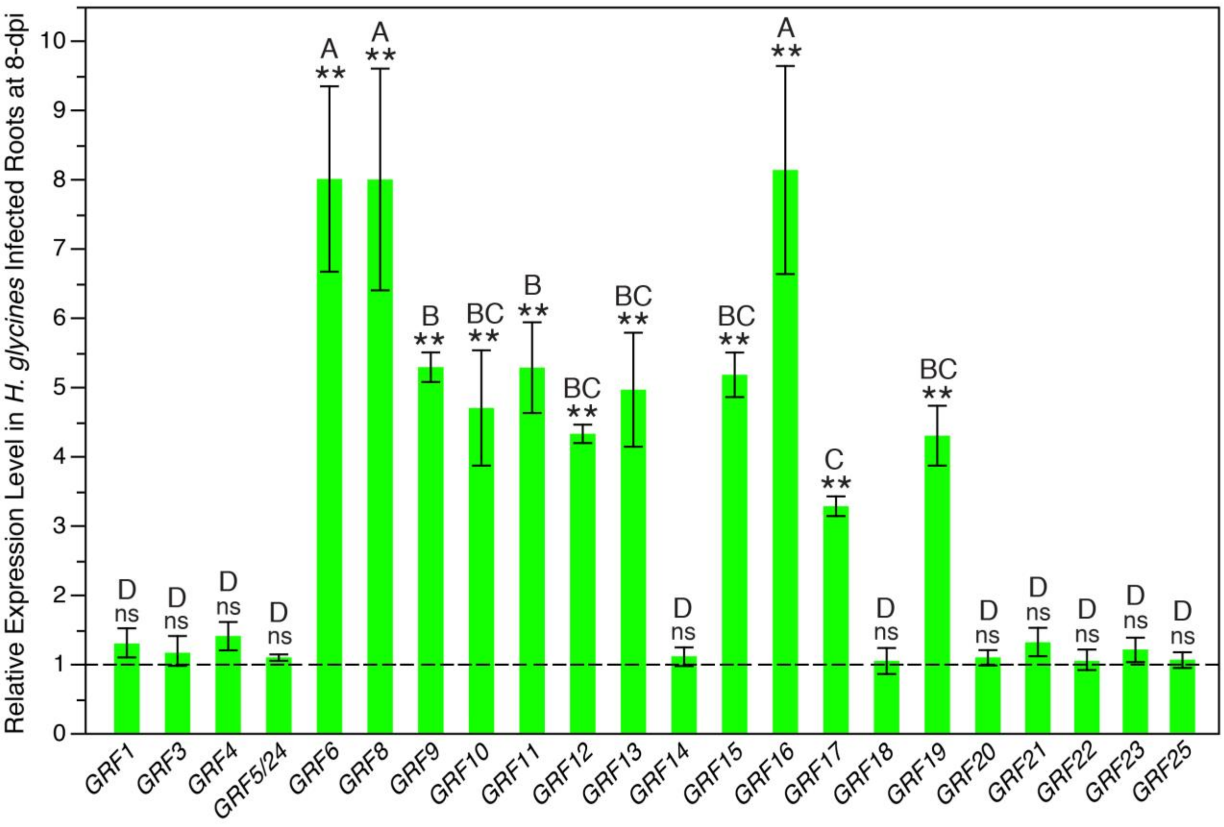

Fig. S4. qRT-PCR screen of *GRFs* in *H. glycines*-infected roots at 8-dpi. Expression levels are relative to mock; baseline expression is set to 1.0 and indicated with a dashed line. Error bars represent ± one standard deviation from the mean. For each *GRF*, the bottom label (ns or **) represents statistical comparisons with mock, and the top label represents statistical cross comparisons between the *GRFs* presented as significance groups ($P < 0.05$). **, $P < 0.01$; ns, not significant ($P > 0.05$).

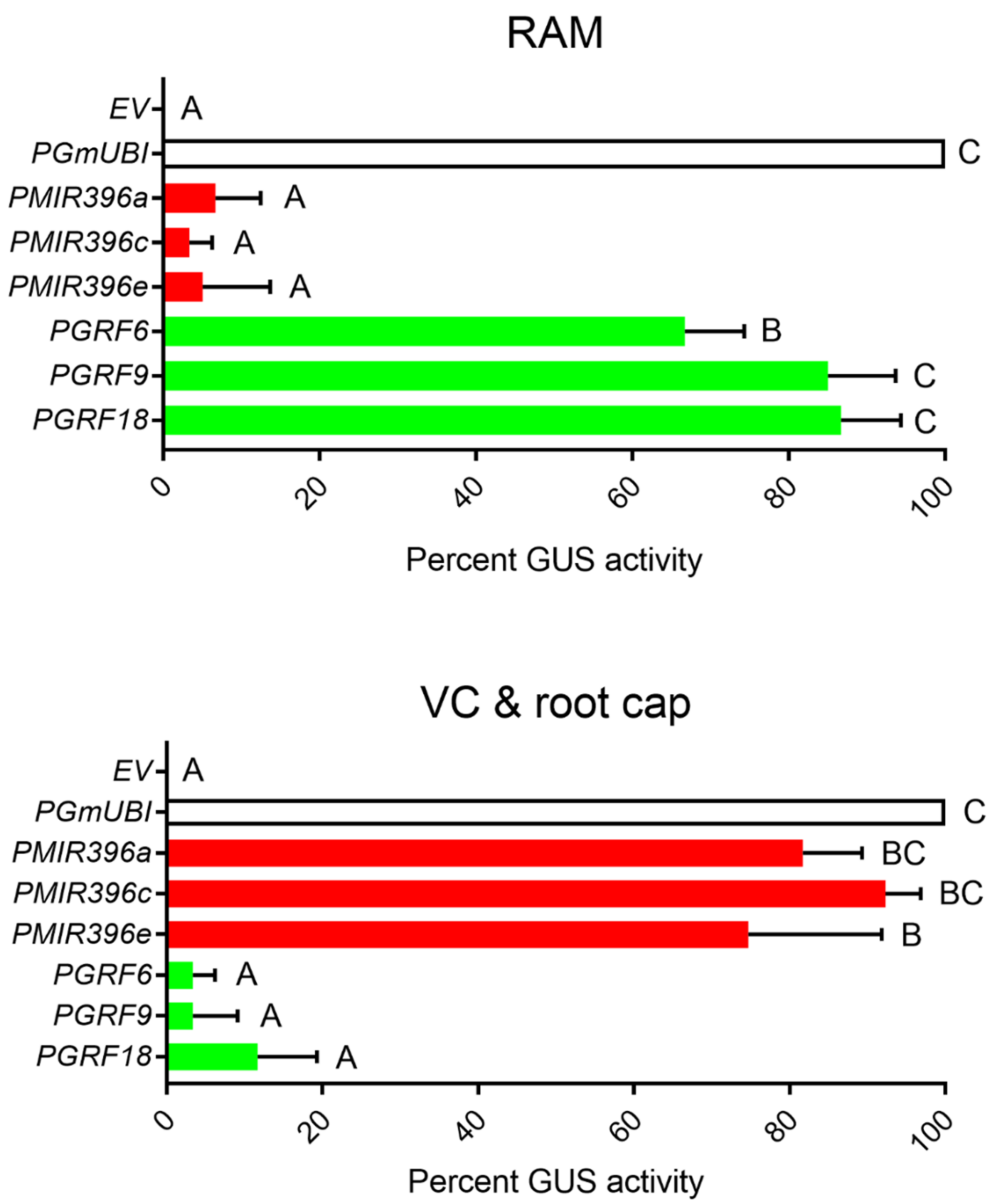

Fig. S5. Activities of selected *MIR396* and *GRF* promoters in *H. glycines*-infected soybean roots outside of syncytia. Shown is the mean percent GUS activity (n=30 infected roots; 10 at 5-dpi, 10 at 8-dpi, 10 at 15-dpi) in the RAM (top), and vascular cylinder and root cap (bottom). Data was pooled from three different experiments. Notice that the activity of these promoters outside of syncytia during infection closely mirrors the uninfected condition (see Fig. 3C,D). Error bars represent ± one standard deviation from the mean. Significance groups are shown at $P < 0.05$.

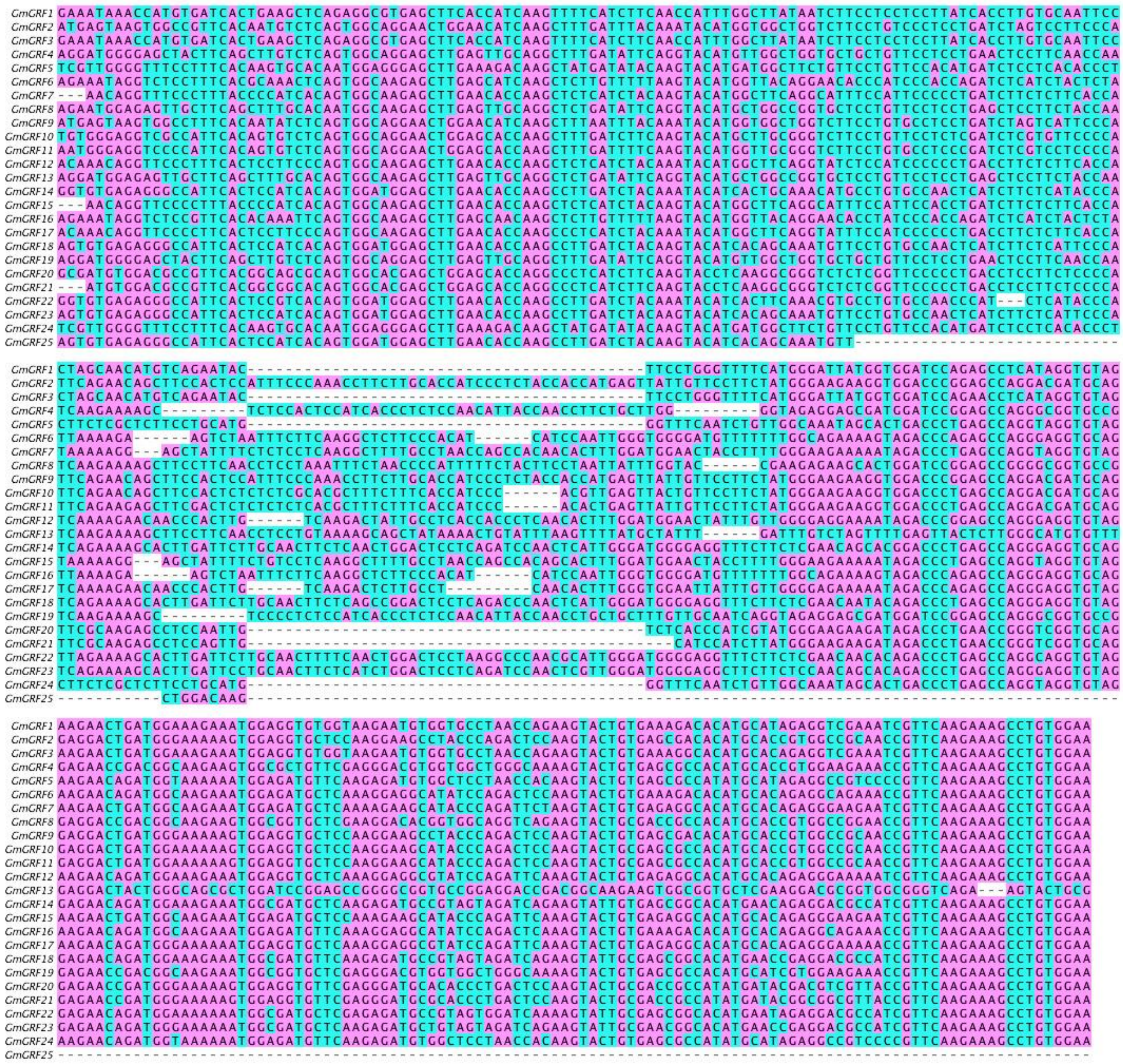

Fig. S6. Multiple sequence (MUSCLE) alignment of soybean *GRFs*.

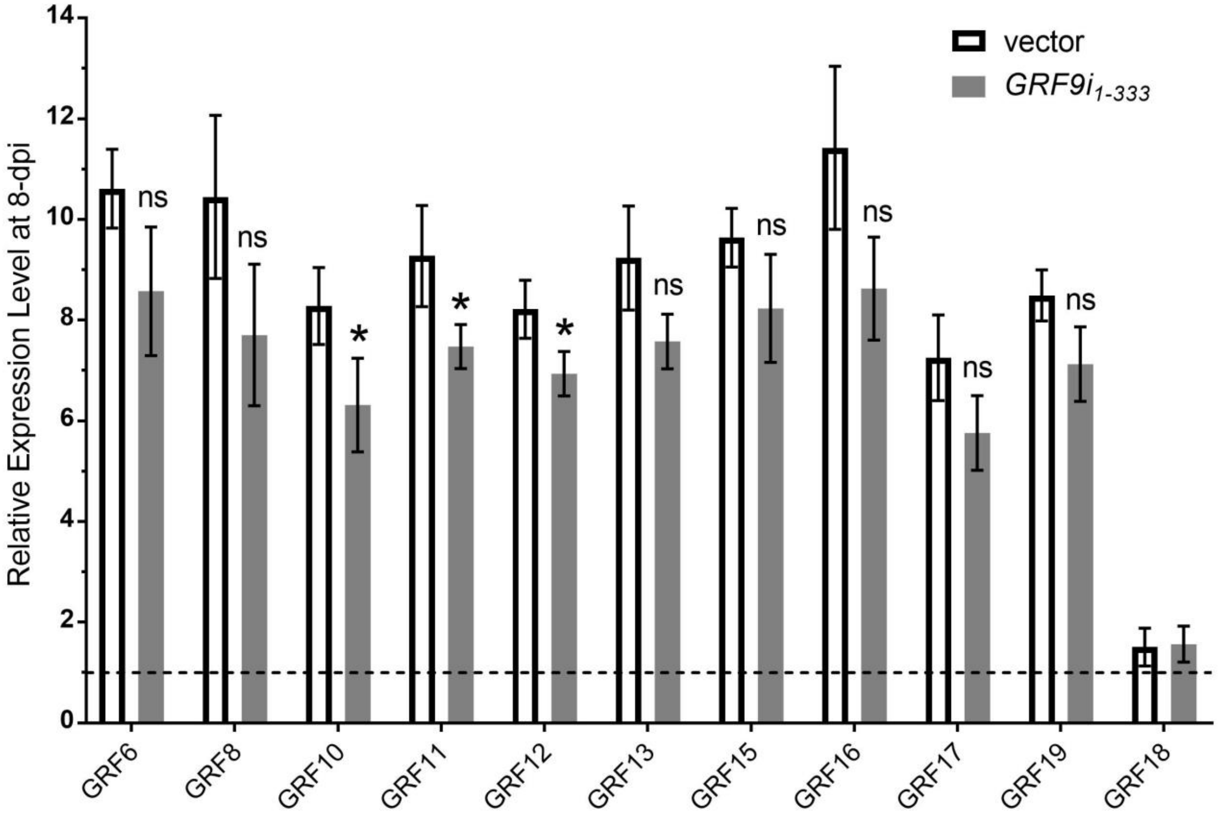

Fig. S7. qRT-PCR analysis of *H. glycines*-responsive *GRFs* in *GRF9i₁₋₃₃₃* roots. The analysis was performed as in (Fig. 4G).

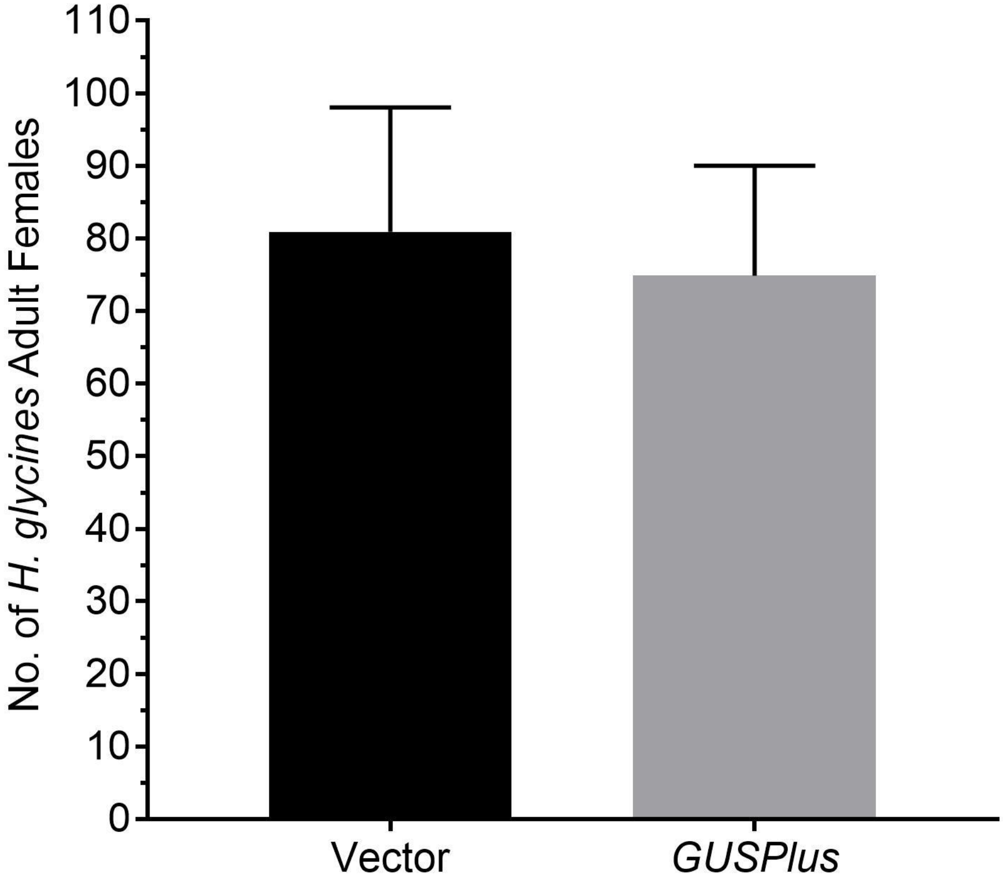

Fig. S8. *PGmUBI*-driven expression of *GUSPlus* does not change susceptibility to *H. glycines* infection. Susceptibility of p4305.1 (*PGmUBI:GUSPlus*) soybean roots to *H. glycines* was compared to pG2XPRESS empty vector control roots (*n* = 20).

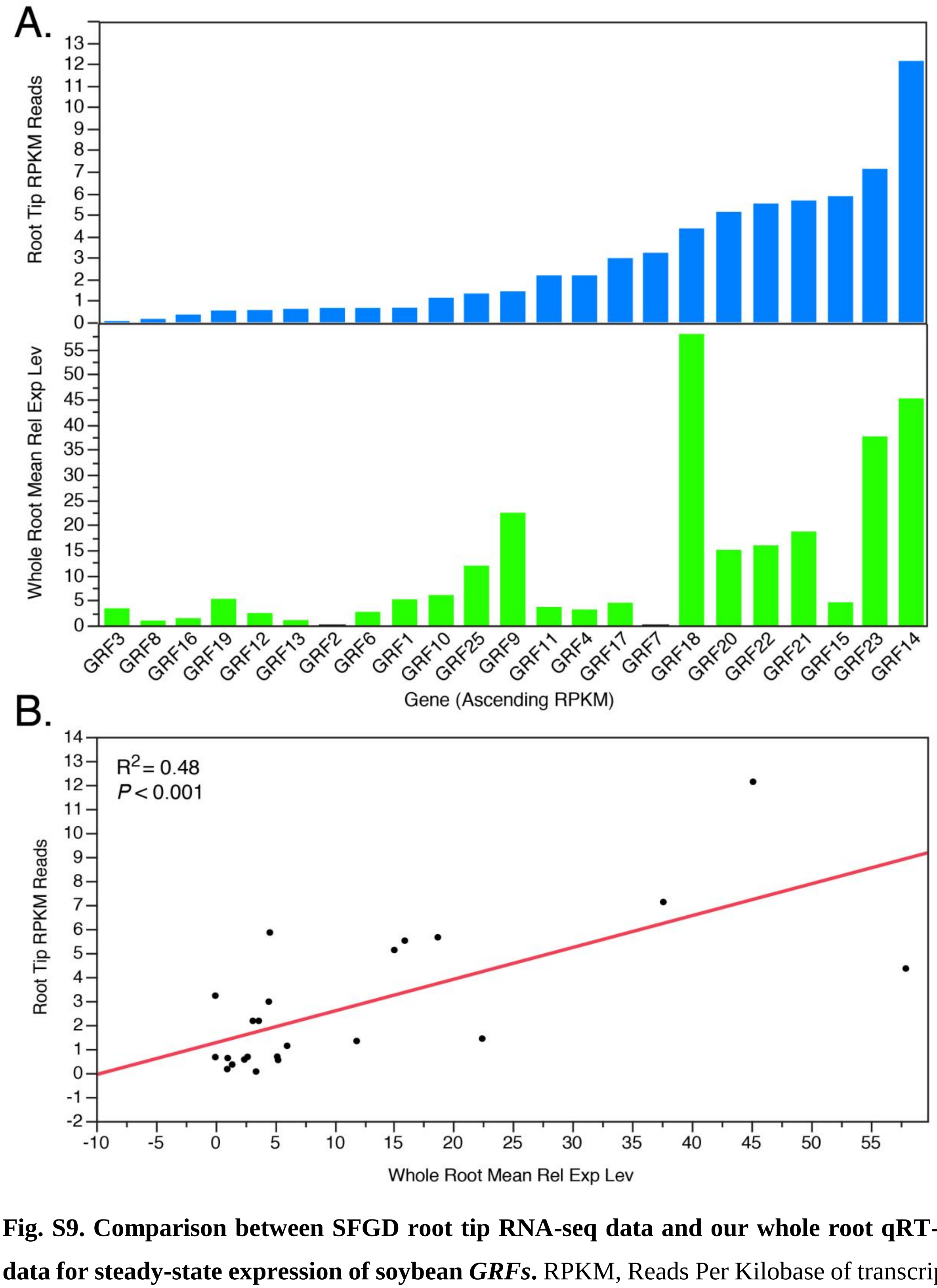

Fig. S9. Comparison between SFGD root tip RNA-seq data and our whole root qRT-PCR data for steady-state expression of soybean *GRFs*. RPKM, Reads Per Kilobase of transcript per Million mapped reads.

Table S1. Complete list of primers used in our study.

Primer ID	Primer Sequence (5'-3')
**pre-miR396a/i_qPCRF**	CTGCATCCAAAGAGTTCCTTTGC
**pre-miR396b/d/k_qPCRF**	AAGTCCTGGTCATGCTTTTCCAC
**pre-miR396c/f_qPCRF**	GTCCTGTTATGCTTTTCCACAGC
**pre-miR396e/h/j_qPCRF**	CTTGAACTGTGTTGTGAGGCTTC
**miR396a/i/j_qPCRF**	TTCCACAGCTTTCTTGAACTGAA
**miR396b/c/d/f/k_qPCRF**	TTCCACAGCTTTCTTGAACTTAA
**miR396e_qPCRF**	TTCCACAGCTTTCTTGAACTGTAA
**GRF1_qPCRF**	ATGAACCGGCAGTTACCATTGCG
**GRF1_qPCRR**	ATGGTTCACTTGCTGGGACTGAC
**GRF2_qPCRF**	TGACTGTAACTGCTGGCAGCAG
**GRF2_qPCRR**	CTGATCCAGAACCAGTCCCTTGG
**GRF3_qPCRF**	CAGCAGCTACTGCTACCAGTTCC
**GRF3_qPCRR**	ATGAATGCTTCCCTGGCTTGGAC
**GRF4_qPCRF**	CTTCGATGGGAGGTCCACTTGC
**GRF4_qPCRR**	TCCACGAGGCAAGTGACGATG
**GRF5/24_qPCRF**	ATCTCCATCTGGGGTGTTGCAG
**GRF5/24_qPCRR**	AGCAAGGCCATCTCAGAATTGGC
**GRF6_qPCRF**	CATGACACCACCCTCTTCCTTCC
**GRF6_qPCRR**	GAAAAGGGTTGGGACTCAATAGGC
**GRF7_qPCRF**	AACAAACACAAACTCAAATGCTTC
**GRF7_qPCRR**	CACCAACACCACCCTCAACACTA
**GRF8_qPCRF**	TGGGGATGGCAAATCAAATGGCC
**GRF8_qPCRR**	TCAACGACACATCCGAGGGATCG
**GRF9_qPCRF**	CACTTTGATGCCAACCGGTGGAG
**GRF9_qPCRR**	GGACTGACCCTCTTGCTTCACAG
**GRF10_qPCRF**	CTCACTGTCACTGGGGGTAGTGG
**GRF10_qPCRR**	CAGTTCCAGAATCGGTACCCTGG
**GRF11_qPCRF**	CTGAGGGTGGTGAACACTGCTTC
**GRF11_qPCRR**	AGAGGCAACTCTGGTTGACATCG
**GRF12_qPCRF**	GCAGCAGTGGCAACAAACACAG
**GRF12_qPCRR**	GGAGAGTGCATGTGCAGGACTG

**GRF13_qPCRF**	GTGGATGGGGATGGAAATTCAGC
**GRF13_qPCRR**	TCAACGACACATCCGAGGAAGTG
**GRF14_qPCRF**	CACAACAAAGTGCACCACCCTC
**GRF14_qPCRR**	GTAGCATGGGAAGGGCAGAGC
**GRF15_qPCRF**	AATAGCACTCTCTCACCTCTTGC
**GRF15_qPCRR**	GAGGATAGTGGTGGTGTTGGTGAG
**GRF16_qPCRF**	CCACCACCACCCTCTTCTTTCCC
**GRF16_qPCRR**	TGGGAAAAGGGTTGGGACTGATC
**GRF17_qPCRF**	CAGTGCCCCCTTGTTTCTCGAC
**GRF17_qPCRR**	CCATACACATACCTGCAGTCAGTG
**GRF18_qPCRF**	TAGGTCATCCCCAACTGGGGTG
**GRF18_qPCRR**	CATCTCTGCTCCCAGCACTGC
**GRF19_qPCRF**	AAAACAATGCTGCTGGCCCTAGC
**GRF19_qPCRR**	GGAAGCAGCAGCAGCATTTCCG
**GRF20_qPCRF**	GTTGCTAATGTTTCTGCAACTGCTGC
**GRF20_qPCRR**	GTGTTGGTGTTGGTGTTGGTGC
**GRF21_qPCRF**	TGTTTCTGGAACTGCAACTGCTGC
**GRF21_qPCRR**	CCCTTGCACCATTGGTGTGC
**GRF22_qPCRF**	GCCGGTGGAAGGTCTTTTCGTGG
**GRF22_qPCRR**	TGGCCCTCTCACACCAGCCA
**GRF23_qPCRF**	AGGCCATGCCCTCACCACCA
**GRF23_qPCRR**	TGTTGTCCTTTGGTTTCAGCACGGT
**GRF25_qPCRF**	TCCAAAGTCAGAGACTTTATTGGTAGAC
**GRF25_qPCRR**	TGCTTATGCTTCCTGTATTTTTGCTGTA
**GmUBQ3_qPCRF**	GACCAGCAACGTCTCATTTTCGC
**GmUBQ3_qPCRR**	GTGTCGGAGCTTTCAACCTCTAGG
**GRF6_5'-RACE_outer**	TTCCATGAACATGCCTGCCA
**GRF6_5'-RACE_inner**	CACATTCAACTCGGTTAAAGACCA
**GRF8_5'-RACE_outer**	GTCCTCTCCATAGCCAGTGGTCA
**GRF8_5'-RACE_inner**	TCACGGTTTCTAGACAAGCTCTTGCTG
**GRF9_5'-RACE_outer**	AGAGCTAGTTGCAGAGAAATTTGACGAT
**GRF9_5'-RACE_inner**	TGGGTTTGGTCTGTTCCAGAAGCAG
**GRF10_5'-RACE_outer**	CACATGTCAGACCATCACCCA
**GRF10_5'-RACE_inner**	TGTGGTCAGTTCCAGAATCGG

**GRF11_5'-RACE_outer**	AAATTGAATCTCATTCTCACCATGT
**GRF11_5'-RACE_inner**	GAAGGCCATGTGTTTTCCAGC
**GRF12_5'-RACE_outer**	CTCTGAAGTTGCAAGTAGGAGTACTCGT
**GRF12_5'-RACE_inner**	TACTTCATCAAGCCAAGCAGGCTTG
**GRF13_5'-RACE_outer**	TCCTGAGTTGTTGTTCTGGCAACCA
**GRF13_5'-RACE_inner**	CCATCCCCATCCACATGATCATGGT
**GRF15_5'-RACE_outer**	TGTCATCCAAATCAAGCCAAGAGCCT
**GRF15_5'-RACE_inner**	GACAAGCCAAAGGAACTCCTCTGCT
**GRF16_5'-RACE_outer**	GGAGCTCATTGATAGTTGTTGGTACGAG
**GRF16_5'-RACE_inner**	CATGAGAATAAGAGCCAGCAGAGTCCA
**GRF17_5'-RACE_outer**	TTGCCACTGAGGCTCTGAAG
**GRF17_5'-RACE_inner**	AAGGCATGCTCATCCACCTC
**GRF19_5'-RACE_outer**	GGAAGCAGCAGCAGCATTTCCG
**GRF19_5'-RACE_inner**	AGATCAAATGGGGATTTGAGTGAAGCAG
**PMIR396c_CLONE_F**	ATTCGT**CTGCAG**GCATCCTTTGCTGCAACTTCAACTC
**PMIR396c_CLONE_R**	TAAGCA**GTCGAC**AAGAAAGCATGGAAGTTGAAATCCTGAAGC
**PGRF9_CLONE_F**	TAAGCA**CTGCAG**AGAGGTGTGGCTTGCAAGGA
**PGRF9_CLONE_R**	TAAGCA**GTCGAC**TCTCCTCCCCCACTCTTCTCTTCTC
**pre-miR396a_CLONE_F**	TAAGCA**ATTTAAAT**TTGTGGGGTATATTCAGTCTTCAATA
**pre-miR396a_CLONE_R**	TAAGCA**GGATCC**TGGAGTTTGAGATAAGCTAGTCCGTT
**pre-miR396b_CLONE_F**	TAAGCA**ATTTAAAT**GAGAGATCTGAGCTCAATTTTCCTCT
**pre-miR396b_CLONE_R**	TAAGCA**GGATCC**GTGATGGTACCAAATGAAAGCAATTAAA
**pre-miR396c_CLONE_F**	TAAGCA**ATTTAAAT**CAGGATTTCAACTTCCATGCTTTCTT
**pre-miR396c_CLONE_R**	TAAGCA**GGATCC**TTGTGTGGGATTTTTTAGAACCAATT
**pre-miR396d_CLONE_F**	TAAGCA**ATTTAAAT**TGAGCTCAATTTTCCTCTCAAGTCCT
**pre-miR396d_CLONE_R**	TAAGCA**GGATCC**ACCAAATTAAGGCAATTAAAAGCCTGAA
**pre-miR396e_CLONE_F**	TAAGCA**ATTTAAAT**GTGTTGCTTTGGGAATGGTCTTTTTC
**pre-miR396e_CLONE_R**	TAAGCA**GGATCC**AGGAATTGATGAACCAGATCATCTCA
**pre-miR396f_CLONE_F**	TAAGCA**ATTTAAAT**CACTCCTCTAACTCAATTCATTCTGC
**pre-miR396f_CLONE_R**	TAAGCA**GGATCC**GGTGTACTGTGTAAGTTCTTATAACCA
**pre-miR396i_CLONE_F**	TAAGCA**ATTTAAAT**TTGTGGAGTATTTATCTTCAGTCTTCA
**pre-miR396i_CLONE_R**	TAAGCA**GGATCC**TTTGAGATAAGCTAGTTCGTTGTTGT
**pre-miR396j_CLONE_F**	TAAGCA**ATTTAAAT**ATTCCGTAGGGTGTTGCTTTGGGA
**pre-miR396j_CLONE_R**	TAAGCA**GGATCC**TCAAAGAGGAATTGATGAACCAGATCATCTC

**GRF9_CLONE_startF**	TAAGCA**GGCGCGCC**ATGAGTAAGTGGCCTTTCACAATATCTC
**GRF9_CLONE_stopR**	TAAGCA**CCTAGG**TTAAATCTCACCATGTGGGGAATGAG
**rGRF9_OEPCR_R**	CTCTACGGGTTTCCTAGAGCGGTTGCGGCCACGGTGCATGTGTCG
**rGRF9_OEPCR_F**	CCGCTCTAGGAAACCCGTAGAGTCACAAACTATGACACAGTCATCATC
**GRF9_RNAi_sense_F**	TAAGCA**ATTTAAAT**ATGAGTAAGTGGCCTTTCACAATATCTC
**GRF9_RNAi_sense_R**	TAAGCA**ATTTAAAT**TTCCACAGGCTTTCTTGAACGG
**GRF9_RNAi_anti_F**	TAAGCA**GGATCC**ATGAGTAAGTGGCCTTTCACAATATCTC
**GRF9_RNAi_anti_R**	TAAGCA**GGATCC**TTCCACAGGCTTTCTTGAACGG

Table S2. Soybase genome coordinates and Gene Calls for *MIR396* and *GRFs*.

Gene	Genome Coordinates and Gene Calls from Soybase	Names in Liu et al. 2017
MIR396a	Gm13: 26338134-26338273 [-]	same
MIR396b	Gm13: 26329931-26330056 [+]	same
MIR396c	Gm13: 43804777-43804893 [+]	same
MIR396d	Gm17: 9053049-9053156 [-]	same
MIR396e	Gm17: 35366545-35366658 [-]	same
MIR396f	Gm15: 556691-556841 [-]	same
MIR396g	Gm17: 9053051-9053201 [-] (matches *MIR396d*)	same
MIR396h	Gm14: 13971419-13971566 [+]	same
MIR396i	Gm17: 9044850-9044984 [+]	same
MIR396j	Gm16: 31162190-31162323 [-]	same
MIR396k	Gm17: 8782362-8782224 [-]	same
GRF1	*Glyma01g34650*	*GRF23*
GRF2	*Glyma01g44470*	*GRF15*
GRF3	*Glyma03g02500*	*GRF24*
GRF4	*Glyma03g35010*	*GRF19*
GRF5	*Glyma04g40880* (nearly identical to *GRF24*)	*GRF9*
GRF6	*Glyma07g04290*	*GRF12*
GRF7	*Glyma09g07990*	*GRF2*
GRF8	*Glyma10g07790*	*GRF21*
GRF9	*Glyma11g01060*	*GRF16*
GRF10	*Glyma11g11820*	*GRF13*
GRF11	*Glyma12g01730*	*GRF14*
GRF12	*Glyma13g16920*	*GRF3*
GRF13	*Glyma13g21630*	*GRF22*
GRF14	*Glyma14g10090*	*GRF6*
GRF15	*Glyma15g19460*	*GRF1*
GRF16	*Glyma16g00970*	*GRF11*
GRF17	*Glyma17g05800*	*GRF4*
GRF18	*Glyma17g35090*	*GRF7*
GRF19	*Glyma19g37740*	*GRF20*
GRF20	*Glyma09g34560*	*GRF17*
GRF21	*Glyma01g35140*	*GRF18*
GRF22	*Glyma14g10100*	*GRF8*
GRF23	*Glyma17g35100*	*GRF5*
GRF24	*Glyma06g13960* (nearly identical to *GRF5*)	*GRF10*
GRF25	*Glyma08g34500*	*GRF26*
		GRF12
